# Tau accumulation and its spatial progression across the Alzheimer’s disease spectrum

**DOI:** 10.1093/braincomms/fcae031

**Published:** 2024-02-07

**Authors:** Frédéric St-Onge, Marianne Chapleau, John C S Breitner, Sylvia Villeneuve, Alexa Pichet Binette

**Affiliations:** Integrated Program in Neuroscience, Faculty of Medicine, McGill University, Montreal, QC H3A 2B4, Canada; Research Center of the Douglas Mental Health University Institute, Montreal, QC H4H 1R3, Canada; Faculty of Medicine, University of California San Francisco, San Francisco, CA 94143, USA; Research Center of the Douglas Mental Health University Institute, Montreal, QC H4H 1R3, Canada; Department of Psychiatry, Faculty of Medicine, McGill University, Montreal, QC H3A 1Y2, Canada; Research Center of the Douglas Mental Health University Institute, Montreal, QC H4H 1R3, Canada; Department of Psychiatry, Faculty of Medicine, McGill University, Montreal, QC H3A 1Y2, Canada; McConnell Brain Imaging Centre, Montreal Neurological Institute, Montreal, QC H3A 2B4, Canada; Clinical Memory Research Unit, Faculty of Medicine, Lund University, Malmö 205 02, Sweden

**Keywords:** tau, spatial extent, Alzheimer’s disease, positron emission tomography

## Abstract

The accumulation of tau abnormality in sporadic Alzheimer’s disease is believed typically to follow neuropathologically defined Braak staging. Recent *in-vivo* PET evidence challenges this belief, however, as accumulation patterns for tau appear heterogeneous among individuals with varying clinical expressions of Alzheimer’s disease. We, therefore, sought a better understanding of the spatial distribution of tau in the preclinical and clinical phases of sporadic Alzheimer’s disease and its association with cognitive decline. Longitudinal tau-PET data (1370 scans) from 832 participants (463 cognitively unimpaired, 277 with mild cognitive impairment and 92 with Alzheimer’s disease dementia) were obtained from the Alzheimer’s Disease Neuroimaging Initiative. Among these, we defined thresholds of abnormal tau deposition in 70 brain regions from the Desikan atlas, and for each group of regions characteristic of Braak staging. We summed each scan’s number of regions with abnormal tau deposition to form a spatial extent index. We then examined patterns of tau pathology cross-sectionally and longitudinally and assessed their heterogeneity. Finally, we compared our spatial extent index of tau uptake with a temporal meta-region of interest—a commonly used proxy of tau burden—assessing their association with cognitive scores and clinical progression. More than 80% of amyloid-beta positive participants across diagnostic groups followed typical Braak staging, both cross-sectionally and longitudinally. Within each Braak stage, however, the pattern of abnormality demonstrated significant heterogeneity such that the overlap of abnormal regions across participants averaged less than 50%, particularly in persons with mild cognitive impairment. Accumulation of tau progressed more rapidly among cognitively unimpaired and participants with mild cognitive impairment (1.2 newly abnormal regions per year) compared to participants with Alzheimer’s disease dementia (less than 1 newly abnormal region per year). Comparing the association of tau pathology and cognitive performance our spatial extent index was superior to the temporal meta-region of interest for identifying associations with memory in cognitively unimpaired individuals and explained more variance for measures of executive function in patients with mild cognitive impairments and Alzheimer’s disease dementia. Thus, while participants broadly followed Braak stages, significant individual regional heterogeneity of tau binding was observed at each clinical stage. Progression of the spatial extent of tau pathology appears to be fastest in cognitively unimpaired and persons with mild cognitive impairment. Exploring the spatial distribution of tau deposits throughout the entire brain may uncover further pathological variations and their correlation with cognitive impairments.

## Teaser

St-Onge *et al*. report that, despite following the classical Braak stages, patterns of tau pathology differ between individuals on the spectrum of typical Alzheimer’s disease, and accounting for that variability improves associations with cognitive domains.

## Graphical Abstract

**Graphical Abstract:**
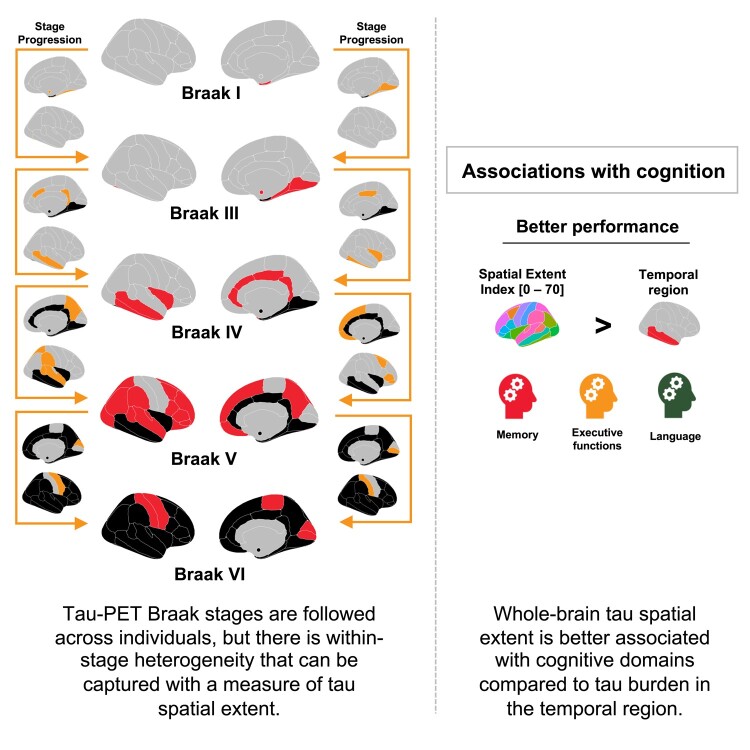

## Introduction

The first PET tracers of tau pathology were developed almost a decade ago.^1^ These tracers have advanced our understanding of the role of tau pathology in aging and Alzheimer’s disease.^2-5^ However, several questions remain, including the spatial progression of the disease across the whole brain. Our principal aim was to provide a comprehensive view and the clinical relevance of cross-sectional and longitudinal tau-PET binding in late-onset sporadic Alzheimer’s disease. Using data from the Alzheimer’s Disease Neuroimaging Initiative (ADNI), we here report the abnormal tau-PET binding patterns in individuals classified as being cognitively unimpaired (CU) or having mild cognitive impairment (MCI) or Alzheimer’s disease dementia. We also report the amount and the spatial extent of tau abnormality across these clinical groups both cross-sectionally and over time. Finally, we describe their association with cognitive impairment.

The progression of tau pathology in the brain is generally believed to follow a stereotypical pattern approximating the Braak stages defined post-mortem, where tau starts accumulating in medial temporal regions (Braak I-II) before accumulating in limbic regions (Braak III-IV) and finally to the whole cortical mantle (Braak V-VI).^6^ Many PET studies have confirmed this pattern *in vivo*,^5,7,8^ and studies investigating associations between tau and clinical variables usually average tau from a predefined set of temporal regions [i.e. a temporal meta-region of interest (ROI)] to approximate the early stages of tau accumulation.^9-11^

Reports in recent years have highlighted the limitations of this homogenous approach as tau progression patterns can differ across individuals^12,13^ and between different disease variants.^14-16^ These inter-individual differences would seem important to track longitudinal changes, and it has been suggested that tau accumulation is better captured when using individualized ROIs.^12,17^ Inter-individual differences in tau pathology may become particularly critical when tracking clinical progression. The evidence thus far highlights that tau, rather than amyloid-beta (Aβ) alone, is a reliable indicator of future clinical progression,^11,18^ and is well associated with cognitive change in the early stages of Alzheimer’s disease.^19-23^ Therefore, if tau patterns and their progression are indeed heterogeneous, it is likely that tracking tau with a single set of regions across participants may misrepresent a significant portion of them.

Leveraging 1370 tau-PET scan visits from 832 ADNI participants across the Alzheimer’s disease spectrum, we characterized the spatial extent of tau pathology across the whole brain (70 brain regions) both cross-sectionally and longitudinally. We summarized these measures by developing a novel index, the spatial extent index. This index accounts for individual differences in tau-PET patterns by evaluating the extent of tau pathology for any single individual across the whole brain. We then evaluated how the spatial extent index related to performance in different cognitive domains. We compared this approach with more traditional measures of Braak staging and tau-PET uptake in a temporal meta-ROI.^9^ We hypothesized that a region-specific analysis of tau-PET abnormality would offer a more useful measure of cognitive impairment than other approaches that rely on tracer uptake in one set of regions across all individuals.

## Materials and methods

### Participants

We used data from ADNI, a multi-site study launched in 2003 as a public-private partnership. The primary goal of ADNI has been to test whether serial MRI, PET and other biological markers and clinical and neuropsychological assessment can be combined to measure the progression of MCI and early Alzheimer’s disease. For up-to-date information, see www.adni-info.org.

We conducted the analyses using ADNI longitudinal data available in May 2022. We included participants who had at least one available tau (flortaucipir) and one Aβ (florbetapir or florbetaben) PET scan, and who had an available diagnostic assessment within 2 years from the tau scan in ADNI3.

### PET acquisition and processing

We used fully preprocessed data from the ADNI consortium. Details on PET acquisition and preprocessing procedures can be found elsewhere (http://adni.loni.usc.edu/methods/documents/). Briefly, for tau-PET, the flortaucipir tracer ([^18^F] AV-1451) was used and images were acquired 75–105 min post-injection. For Aβ-PET, florbetapir or florbetaben were used, and images were acquired 50–70- and 90–110-min post-injection, respectively. Briefly, PET images were realigned, averaged, resliced to 1.5 mm^3^ and smoothed to a resolution of 8 mm^3^ full width at half-maximum. Then, the closest T1-weighted MRI available for a participant was processed and segmented using FreeSurfer 7.1.1, and co-registered to the PET scan using statistical parametric mapping. Standardized uptake value ratios (SUVRs) were extracted from each cortical region of the Desikan atlas.^24^ The inferior cerebellum was used as the reference region for flortaucipir, and the whole cerebellum was the reference region for Aβ-PET. As suggested by the ADNI PET core group, we divided the SUVR values provided by ADNI by the SUVR values in the reference region for each tracer.

Aβ-PET positivity status was determined according to the cutoff derived from the ADNI PET core based on a neocortial composite region: participants exceeding 1.11 SUVR for florbetapir or 1.08 SUVR for florbetaben were considered positive. We also converted the SUVR values into centiloid units for supplementary analyses, following established formulas from the ADNI PET core.^25^

### Regional tau-PET and other measures of interest

**Figure 1:**
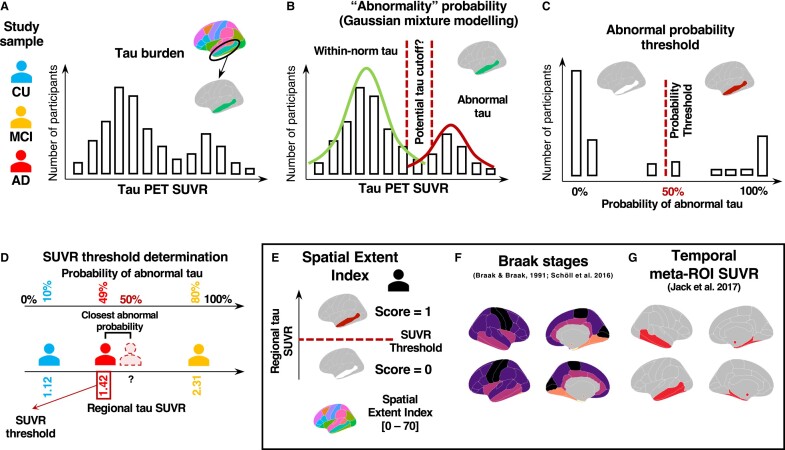
**Spatial extent methodology.** For each cortical region of the Desikan atlas and the bilateral amygdalae, we extract the SUVR of our participants (**A**). Then, a two-component Gaussian mixture modelling technique is applied to the SUVR values in each region (**B** and **C**). The second distribution is considered to reflect abnormally high SUVR tau values. We extract the probability that each participant belongs to the ‘abnormal’ distribution and establish a threshold that individuals with over 50% probability are considered positive for the given region (**D**). Once thresholds are derived across all regions, we derive the spatial extent index for each participant by summing the number of positive regions across the brain (**E**). We also apply the same methodology to the average SUVR within each aggregate composing Braak stages I and III through VI (**F**). To compare our spatial extent index in the cognition analyses, we also compute the average SUVR in a classic temporal meta-ROI. (**G**). Figure adapted from *sihnpy*’s documentation (https://sihnpy.readthedocs.io/), available with a CC-BY licence.

Our main interest was to study the patterns of elevated regional tau-PET uptake across the brain at the individual level. For this aim, we derived an SUVR cutoff for each brain region of interest using Gaussian mixture modelling (GMM) on the entire cross-sectional sample of ADNI participants. This procedure is illustrated in Fig. 1. We fitted a two-component GMM for each region and used the SUVR closest to the 50% probability of belonging to the abnormal (high values) distribution as the regional cutoff, as done previously.^26,27^ The GMMs were initialized using k-means and parametrized using scikit-learn’s v1.2.1 default settings. We ensured that a two-component solution was a better fit compared to a single-component solution by verifying that the Bayesian information criterion of the two-component solution was higher. The brain regions of interest were the 34 bilateral cortical regions of the Desikan atlas^24^ and the amygdalae. We then binarized the tau SUVR from each region, and values at or exceeding the cutoff were coded as one and a score lower than the cutoff as zero. From there, we derived our main measure of interest: the spatial extent index, which is the sum of regions exceeding the regional thresholds for a given participant. Regional thresholds for each region are provided in Supplementary Table 1. The main results were replicated by setting regional thresholds based on 2 SD from the mean of the tau SUVR of CU Aβ− participants and deriving the spatial extent with these alternative thresholds (see Supplementary Results, Supplementary Tables 2 and 5 and Supplementary Figs. 9–13).

We also derived a more typical temporal meta-ROI^9^ and the regions composing the Braak staging scheme.^5,6,8^ The temporal meta-ROI was the average SUVR from key regions harbouring elevated tau-PET SUVR in Alzheimer’s disease: the entorhinal cortex, the parahippocampal, inferior temporal, the middle temporal and fusiform gyri and the amygdalae.^9^ In the Braak staging scheme, pathology accumulation follows a predetermined order ranging from Braak I to VI until the whole cortical mantle is affected by tau (see Supplementary Table 1 for all regions included in each stage).^6,8^ Braak II (hippocampus) was excluded from our analyses owing to the known choroid plexus off-target binding effect of the flortaucipir tau-PET tracer.^3^ We averaged the tau-PET SUVR values in bilateral regions comprising each Braak stage, following methods described previously.^5,28^ We then applied the GMM approach, as described in Fig. 1, to determine a data-driven threshold for each Braak stage. These thresholds were then applied to assign which individuals were positive on each Braak stage.

A subset of 195 participants had at least two tau-PET scans for longitudinal analyses, with 100 having three such scans. The same regional binarization of positive (score 1) or negative (score 0) using the regional cutoffs was applied to all time points.

### Neuropsychological measures

To compare the clinical implications of our regional index score versus a typical meta-ROI analysis, we compared the association of each with composite cognitive scores for memory, executive function,^29^ language and visuospatial performance.^30^ The cognitive performance data were taken as the test time point closest in time to tau-PET. As well, we assessed cognitive decline in participants by estimating slopes of annual change for each cognitive composite score using linear mixed-effects models with random slopes and intercepts. For these analyses, the cognitive score at each visit was the outcome, with the exposure being time since the initial cognitive test score in ADNI. These analyses considered all ADNI visits for the whole sample, thereby maximizing the number of time points contributing to estimates of individual slopes. For all cognitive domains, models met assumptions of linearity, homoscedasticity, and normality of residuals, except for the visuospatial score, where very small change over time was observed.

### Statistical analyses

All statistical analyses were run using Python v3.9.2 (numpy v1.23.1; pandas v1.4.3; scipy v1.9.3; scikit-learn v1.2.1; matplotlib v3.6.3), R v4.2.0 (Packages: lme4 v1.1-30; tidyverse v1.3.1; lmerTest v3.1-3; lmtest 0.9–40; nonnest2 v0.5-5; tableone v0.13.2; patchwork v1.1.2; ggseg v1.6.5; ggnewscale v0.4.7; glue v1.6.2; MASS v7.3-59; cocor v1.1-4; performance v0.10.1; pscl v1.5.5.1) and R Studio ‘Prairie Trillium’ Release (1db809b8, 2022-05-16) for macOS.

#### Demographics

We compared groups on their demographic information by their diagnostic status separately for Aβ+ and Aβ− participants using one-way ANOVA and Tukey *post hoc* tests being used for continuous variables and chi-square tests for categorical variables.

#### Cross-sectional characterization of tau

We first compared tau levels of Aβ-positive versus Aβ-negative individuals. For the three diagnostic groups of CU, MCI or Alzheimer’s disease dementia, we compared our spatial extent index with the temporal meta-ROI SUVR contrasting Aβ+ and Aβ− individuals within each clinical group using ANOVA and *post hoc* Tukey tests. Logistic regression complemented this analysis by quantifying the probability of having a spatial extent index of at least one based on a continuous burden of Aβ pathology (centiloid values). The linearity of log odds of having a spatial extent of at least one to centiloid values was verified. As tau-PET binding was typically low in Aβ− participants, all subsequent analyses were done separately in each diagnostic group in the Aβ+ sample. We calculated the extent to which each participant’s tau pathology was consistent with Braak staging. To do this, at each Braak stage, we computed the percentage of participants who were tau-positive both at their more advanced Braak stage and at all previous stages (e.g. if a participant was positive on Braak IV, and was also positive on Braak III and I, then this participant was judged to have data in accord with Braak staging).

#### Longitudinal characterization of tau

We used linear mixed-effect models to calculate the annual change of the tau spatial extent and the temporal meta-ROI (tau as the outcome; time since first tau scan as exposure) with random slopes and intercepts for each participant for the temporal meta-ROI. As the spatial extent index represents a count of regions, we used a Poisson mixed model to model the longitudinal change correcting for zero inflation as the models significantly underfitted the zero counts. At the group level, we used linear mixed-effect models with random slopes and intercepts to track the annual change in positivity across the cohort and the annual change in SUVR in each brain region and plotted the regions on a template brain map. We calculated the extent to which Braak stages were followed by participants longitudinally. For each Braak stage, we computed the percentage of participants who became positive at each stage, and who were already positive or progressed in the previous Braak stages (e.g. if a participant became positive on Braak IV at their last visit and was already positive or progressed in Braak III and I, the participant followed the Braak stages).

#### Tau-PET heterogeneity

We computed the overlap between the patterns of abnormal tau at baseline or over time between participants in the same diagnostic group using the Jaccard similarity index. The index ranges from zero to one where zero indicates that not a single positive region overlaps between participants, and one indicates that all positive regions between two participants perfectly overlap. We then averaged the values so that each participant would be left with a single value representing, on average, how similar their tau positivity pattern was to the rest of their diagnostic group at the whole brain level. Analyses were always restricted to individuals with at least one positive region.

#### Associations with demographic variables and cognition

We assessed whether the spatial extent index was associated with demographic characteristics (i.e. age, gender, education and *APOE*4 genotype) using linear models, controlling for the other three factors. Then, we studied the association between our tau spatial index measures at baseline and the cognitive performance at the time of the PET, and the cognitive decline (slope) across all available cognitive visits using linear models. *β*, standardized *β*, *P-*values and model fit (*R*^2^ and Akaike information criterion), are reported. Models were adjusted for age, sex and education and were also subjected to a false discovery rate (FDR) multiple comparison correction. Differences in model fit between different tau measures were assessed using Vuong’s closeness test (i.e. non-nested likelihood ratio test).^31^ In complementary analyses, we also assessed the association between tau uptake and cognitive performance in each of the 70 brain regions. Tau SUVR in each region was associated with cognitive performance and cognitive decline for each diagnostic group, controlling for age, sex and education. Within each group, an FDR correction was applied.

## Results

### Participants

A total of 1370 tau scans from 832 unique participants had at least one Aβ and tau-PET scan. At the time of the baseline tau scan, 463 participants were CU, 277 had MCI and 92 had Alzheimer’s disease dementia. About half of the sample (51%) was female, and 34% had at least one *APOE*4 allele. Participants were on average 73.56 ± 7.95 years old. Overall, 35.1% (*n* = 107) of CU individuals, 47.7% (*n* = 132) of individuals with MCI and 83.7% (*n* = 77) of individuals with AD were Aβ-positive. Full demographic information is available in Table 1.

**Table 1:** Demographic information

	Aβ−negative (*n* = 460)			Aβ−positive (*n* = 372)		
	CU (*n* = 300)	MCI (*n* = 145)	AD (*n* = 15)	CU (*n* = 163)	MCI (*n* = 132)	AD (*n* = 77)
Sex, *n* Females, (%)	176 (58.67)	56 (38.62)	5 (33.33)	96 (58.90)	65 (49.24)	32 (41.56)
*APOE*4 carriers, *n* (%)	66 (22.00)	23 (15.86)	5 (33.33)	73 (44.79)	70 (53.03)	48 (62.34)
Age (years)	71.48 (7.31)	73.72 (8.48)	73.83 (8.43)	74.82 (7.57)	74.36 (7.39)	77.35 (8.93)
Education (years)	16.83 (2.30)	16.32 (2.74)	16.07 (2.60)	16.64 (2.34)	15.99 (2.49)	15.55 (2.48)
Centiloid values	4.09 (8.11)	1.18 (10.53)	1.63 (11.27)	53.47 (30.83)	75.78 (35.15)	90.14 (32.86)
Memory composite score	1.08 (0.61)^b,c^	0.52 (0.62)^a,c^	−0.55 (0.48)^a,b^	1.00 (0.62)^b,c^	0.07 (0.59)^a,c^	−0.77 (0.57)^a,b^
Executive composite score	1.20 (0.82)^b,c^	0.61 (0.82)^a,c^	−0.47 (0.95)^a,b^	0.92 (0.77)^b,c^	0.19 (0.92)^a,c^	−0.79 (1.16)^a,b^
Language composite score	0.89 (0.51)^b,c^	0.52 (0.50)^a,c^	−0.21 (0.39)^a,b^	0.75 (0.49)^b,c^	0.41 (0.55)^a,c^	−0.18 (0.61)^a,b^
Visuospatial composite score	0.13 (0.29)^b^	0.01 (0.34)^a^	−0.07 (0.44)	0.06 (0.36)^c^	0.00 (0.38)^c^	−0.43 (0.72)^a,b^
**Longitudinal sub-sample**	**CU** **(*n* = 96)**	**MCI** **(*n* = 40)**	**AD** **(*n* = 10)**	**CU** **(*n* = 90)**	**MCI** **(*n* = 66)**	**AD** **(*n* = 39)**
Average number of tau-PET scan per participant	2.56 (0.87)	2.52 (0.72)	2.20 (0.42)	2.68 (0.75)	2.58 (0.66)	2.54 (0.60)
Average number of cognitive visits per participant	6.24 (3.45)	8.75 (5.59)^a,c^	3.80 (3.88)	6.18 (3.83)	5.55 (4.21)	4.54 (4.02)

^a^significantly different from the CU group.

^b^significantly different from the MCI group.

^c^significantly different from the AD group. Values correspond to mean (standard deviation) unless otherwise specified. *APOE*4 positivity corresponds to having at least one e4 allele. Statistical tests were performed within each of Aβ-negative and Aβ-positive groups.

**Figure 2:**
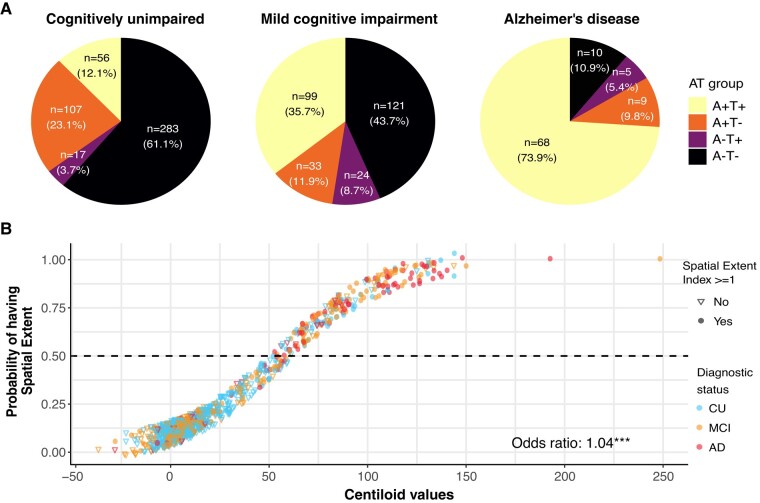
**Amyloid and tau status in the cohort.** (**A**) Aβ/tau status in the included participants from ADNI. Aβ positivity was established using ADNI’s tracer-specific recommendations for both florbetapir and florbetaben. Tau positivity was defined as having at least one region positive for tau pathology (spatial extent index of one and above). (**B**) Scatterplot of the probability of having at least one positive tau region (i.e. spatial extent index equal to or higher than one) as a function of the Aβ load (in centiloid). The probability was extracted from a logitistic regression. The odds ratio (and confidence interval) derived from a logistic regression is presented at the bottom of the graph. Note that the points were jittered by a factor of 0.065 × 0.065 for visualization purposes.

**Figure 3:**
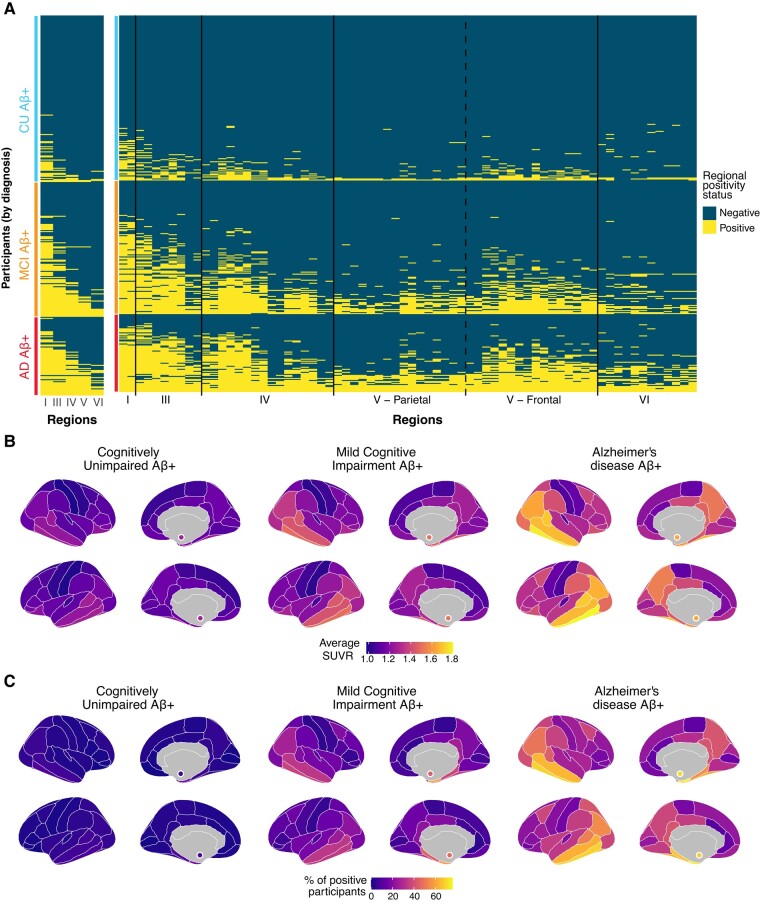
**Spatial extent of abnormal tau deposition in amyloid-positive participants of the ADNI cohort.** (**A**) Based on the method discussed in Fig. 1, abnormality thresholds were determined for each I Braak stages (except stage II) and for each II region of the cortical mantle and the bilateral amygdalae (70 regions). One row on the heatmap corresponds to an individual participant, while each column represents a distinct cortical region. Within each diagnostic group, participants were sorted from individuals with the lowest to highest spatial extent index. Regions on the *x*-axis in II are sorted by Braak stages. (**B**) Regional average SUVR, by diagnostic status. (**C**) Brain maps representing the percentage of participants having abnormal levels of tau in each region, by diagnostic status.

In the Aβ-positive sample, 12.1% (*n* = 56) of CU participants, 36.1% (*n* = 100) of MCI and 73.9% (*n* = 68) of Alzheimer’s disease patients had at least one region of tau positivity (Fig. 2A, heatmap in Fig. 3A). In the Aβ-negative sample, a small percentage of participants had at least one tau-positive region (heatmap in Supplementary Fig. 1B and Supplementary Fig. 2B and C) and had lower tau SUVR in the temporal meta-ROI (Supplementary Fig. 2). Every increase of one Aβ centiloid unit increased the odds of having at least one brain region with abnormal tau tracer uptake abnormal by 4% (Fig. 2B). Considering these findings, and our focus on tau pathology, we restricted the rest of the main analyses to Aβ-positive individuals (*n* = 372).

### Cross-sectional tau-PET patterns

We found that, across diagnostic groups, the entorhinal cortex (Braak I) was the region most positive across Aβ-positive individuals (CU = 17.2%, MCI = 59.9%, Alzheimer’s disease = 74.7%; Fig. 3B, Supplementary Fig. 3 and Supplementary Table 3). In all diagnostic groups, the five regions that were most often tau-positive after the entorhinal cortex were, in order, the inferior temporal (Braak IV), the amygdalae (Braak III), the parahippocampal gyri (Braak III), the middle temporal (Braak IV) and the fusiform gyri (Braak III). All the regions above constituted the temporal meta-ROI.^9^ Similarly, we found that participants largely follow the Braak staging scheme (Fig. 3A): across all Braak stages up to and including Braak V, over 91% of participants positive on any given Braak stage were also positive on all previous Braak stages.

### Longitudinal tau-PET patterns

We repeated the analyses in our longitudinal sample (*n* = 195). Specifically, we assessed whether participants becoming positive in a Braak stage at their last tau scan were either already positive in preceding Braak stages or also progressed in previous stages during the follow-up period.

**Figure 4:**
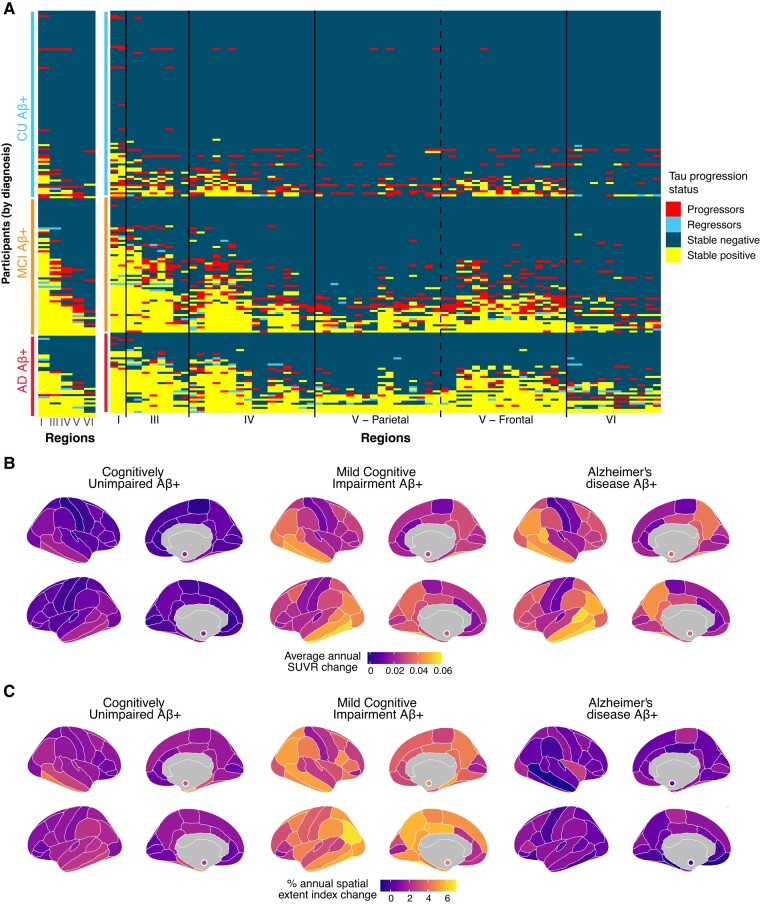
**Spatial localization of abnormal tau accumulation over time in amyloid-positive participants of the ADNI cohort.** (**A**) Abnormal accumulation is presented by (I) Braak stages and (II) all 70 individual brain regions of the Desikan atlas. Colours denote the change in the region between the baseline and the last available visit. A stable region (negative or positive) did not change status during the follow-up. A progressing region was originally negative and subsequently became positive over time. A regressing region was originally positive and became negative over time. (**B**) Brain maps presenting the average SUVR change per region per year. (**C**) Brain maps representing the percentage of participants becoming tau-positive in each region annually. In both **B** and **C**, values in the bilateral amygdalae are represented by small coloured circles in the medial view of the brain, and the annual change is calculated in each region using linear mixed-effect models with random slopes and intercepts. Only participants with at least three tau scans (*n* = 100) were kept for **B** and **C** to ensure a constant sample across the longitudinal follow-ups.

We quantified which brain regions were negative at baseline and became positive over time (progressor), were positive at baseline and became negative over time (regressor), were positive at both visits (stable positive) or were negative at both visits (stable negative). Similar to the cross-sectional results, we found that participants largely followed the Braak staging scheme (Fig. 4A): across all Braak stages up to and including Braak V, over 80% of participants who progressed on a Braak stage at follow-up were already positive or progressed on all previous Braak stages.

Patterns of progression across the brain however were different between clinical stages (Fig. 4; Supplementary Table 4). Specifically, CU participants mostly progressed in the entorhinal cortex (Braak I) while tau abnormality in participants with MCI progressed across the entire cortex, and few participants with Alzheimer’s disease dementia accrued additional tau abnormal regions (Fig. 4C). Based on the tau spatial extent, the annual rate of regions progressing from negative to positive was 1.2 region per year in participants with MCI, which was similar to CU (1.3 region/year) but higher than participants with Alzheimer’s disease dementia (0.988 region/year) (Supplementary Fig. 4B). CU and MCI showed a significant rate of change over time compared to participants with Alzheimer’s disease dementia, which was similar in the temporal meta-ROI.

Few regressions from positive to negative were observed. In terms of Braak stages, four participants with MCI and three participants with Alzheimer’s disease dementia (4% of total participants) regressed from a Braak positive to a negative status (usually Braak III, V or VI). In most cases, the participants only regressed on a single Braak stage. At the regional level, 30 participants (15%) had at least one individual region regressing from positive to negative. The rate of regression was lower in CU (3%) and participants with MCI (18%) compared to participants with Alzheimer’s disease dementia (38%), which could be explained by the higher number of positive regions in these participants.

Overall, we found that participants overwhelmingly followed the Braak staging scheme, demonstrated cross-sectionally and longitudinally, except for the very last Braak stage. However, we also show that there are substantial individual differences in abnormal regions at baseline and in the regional progression of tau pathology.

### Heterogeneity of regional tau abnormality

While abnormal tau accumulation followed Braak staging, regional tau abnormality across the whole brain and within each Braak stage showed heterogeneity across individuals (Fig. 3A; Supplementary Table 2). CU participants demonstrated the least heterogeneity with an average overlap of 0.74 (± 0.15), participants with MCI had an average overlap of 0.58 (± 0.14) and participants with Alzheimer’s disease dementia demonstrated the most heterogeneity with an average overlap of 0.46 (± 0.08). Within each Braak stage, the difference in heterogeneity was greatest between CU and MCI, with the MCI group showing more heterogeneity in the pattern of tau abnormal regions. The difference between MCI and AD was less pronounced and often not significant (Supplementary Fig. 5A). Results were also similar when considering heterogeneity in the progression of regional tau abnormality over time (Supplementary Fig. 5B).

### Associations with demographic information, cognitive profiles and cognitive decline

Given the heterogeneity in regions showing tau abnormality at the individual level across the AD continuum, we then evaluated if the measure of tau spatial extent could yield stronger associations with demographics and cognitive measures than the classical temporal meta-ROI. Of note, the temporal meta-ROI SUVR and the spatial extent correlated well with each other, showing the highest correlation in the MCI group, followed by Alzheimer’s disease and CU (Supplementary Fig. 2C).

Younger participants with MCI (spatial extent index: *β*_std_ = −0.22, *P* < 0.05, *R*^2^_adj_ = 0.08; temporal meta-ROI SUVR: *β*_std_ = −0.20, *P* < 0.05, *R*^2^_adj_ = 0.07) or Alzheimer’s disease dementia (spatial extent index: *β*_std_ = −0.65, *P* < 0.001, *R*^2^_adj_ = 0.39; temporal meta-ROI SUVR: *β*_std_ = −0.46, *P* < 0.001, *R*^2^_adj_ = 0.17) had higher spatial extent index and temporal meta-ROI SUVR (Supplementary Fig. 6). We also found that *APOE*4 carriers in the MCI group had greater tau levels (*β*_std_ = 0.41, *P* < 0.05, *R*^2^_adj_ = 0.09 for the spatial extent index; *β*_std_ = 0.49, *P* < 0.05, *R*^2^_adj_ = 0.10 for temporal meta-ROI SUVR). Sex and education were not associated with the spatial extent index or the temporal meta-ROI.

**Figure 5:**
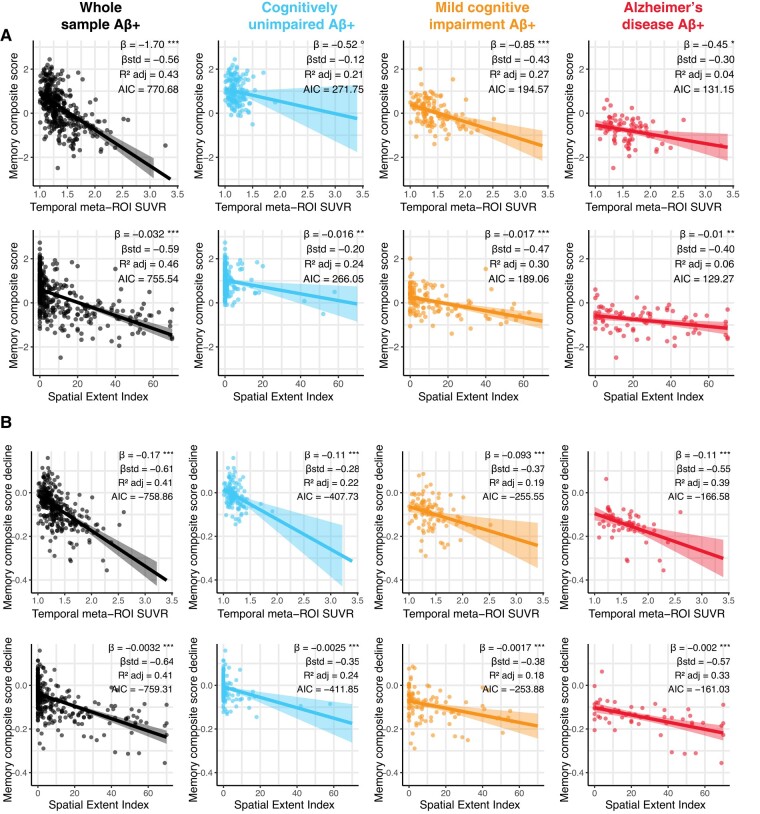
**Association between tau-PET measures, and memory performance and decline.** (**A**) Memory performance closest in time to the tau-PET scan and (**B**) memory decline computed across the study period were associated with both temporal meta-ROI SUVR and spatial extent index in Aβ-positive participants using linear regressions. Cognitive decline was computed for each participant with more than two cognitive time points using linear mixed-effect models with random slopes and intercepts. In each panel, columns represent a diagnostic group (leftmost: whole sample, second from the left: cognitively unimpaired, second from the right: mild cognitive impairment, right-most: Alzheimer’s disease). Simple and standardized *β* coefficients, *adjusted R*^2^ and Akaike information criterion, controlled for age sex and education, are shown on the graphs. *P*-value of models are indicated next to the simple beta coefficients. (°: *P* < 0.1, *: *P* < 0.05, **: *P* < 0.01, ***: *P* < 0.001) Results remained significant after a multiple comparison FDR correction.

In CU participants, the spatial extent index was associated with the memory composite score [*standardized (std) β* = −0.20, *P* < 0.01, *R^2^_adj_* = 0.24] (Fig. 5) while the temporal meta-ROI was not (*std β* = −0.12, *P* > 0.10, *R*^2^*_adj_* = 0.21). The difference in model fit was not significant (*Vuong’s z* = −1.13, *P* = 0.13), however, suggesting that the spatial extent index provided only a marginally better model fit when compared to the more traditional temporal meta-ROI. In CU participants, neither the spatial extent index nor the temporal meta-ROI was associated with any other cognitive composite (executive, language or visuospatial) (Supplementary Figs. 7–9). In participants with MCI, both the spatial extent index and the temporal meta-ROI were nearly equally associated with the memory composite, and there were no differences in model fit (*Vuong’s z* = −1.35, *P* = 0.089). However, the association of the executive composite (*Vuong’s z* = −2.77, *P* = 0.003), as well as the language composite (*Vuong’s z* = −1.89, *P* = 0.029) with the spatial extent index was stronger than that with the temporal meta-ROI. There was no association between the spatial extent index or the meta-ROI and the visuospatial composite. In participants with Alzheimer’s disease, results were similar to participants with MCI: spatial extent index and temporal meta-ROI were both equally associated with the memory, and the spatial extent index was more strongly associated with the executive composite than the temporal meta-ROI (*Vuong’s z* = −1.88, *P* = 0.030). However, the spatial extent index was not more strongly associated with the language composite compared to the temporal meta-ROI SUVR (*Vuong’s z* = 0.04, *P* = 0.516). There was no association between the spatial extent index or the temporal meta-ROI and the visuospatial composite. Looking at cognitive decline, the spatial extent index was more strongly associated with executive function decline compared to the temporal meta-ROI SUVR in participants with MCI (*Vuong’s z* = −1.695, *P* = 0.045). In all other cognitive domains, the temporal meta-ROI and spatial extent index offered a similar model fit for cognitive decline.

**Figure 6:**
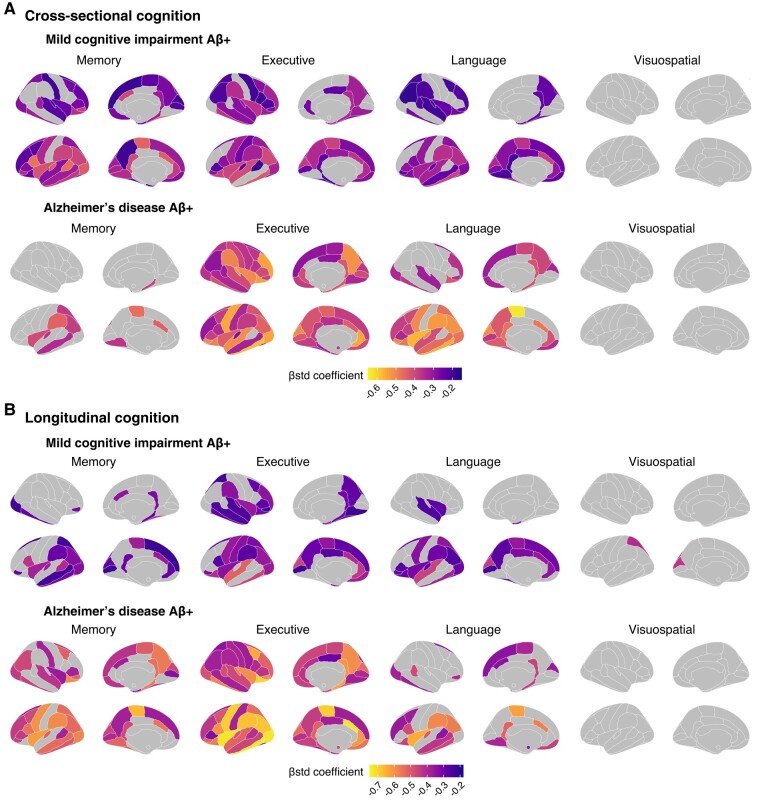
**Region-wise associations between regional tau-PET SUVR and cognitive performance and decline in participants with MCI and Alzheimer’s disease.** Association between tau-PET SUVR and cognitive performance (**A**) and cognitive decline (**B**) in participants with MCI and with Alzheimer’s disease across four cognitive domains (memory, executive functioning, language and visuospatial). Cognitive decline was computed for each participant with more than two cognitive time points using linear mixed-effect models with random slopes and intercepts. The standardized *β* coefficients of the associations between tau-PET SUVR in a specific region and each cognition measure are displayed if it survives adjustment for age, sex and education and a multiple comparison FDR correction (*P_corrected_* < 0.05).

In supplementary analyses, we also investigated regional associations between tau-PET SUVR and cognition (Fig. 6A). In CU participants, no individual region was associated with cognitive performance on any composite score. In participants with MCI, tau levels most strongly associated with memory were in regions of the temporal lobe, with some weaker associations in the parietal and frontal lobes. Tau levels most associated with executive functions comprised regions across the cortex. Associations with language included more regions in the left hemisphere. No associations survived multiple corrections for the visuospatial composite. Results were similar for participants with Alzheimer’s disease dementia.

Looking at the association between baseline tau and longitudinal cognitive decline, region-wise analyses between tau SUVR and cognitive decline largely replicated our findings at the cross-sectional level (Fig. 6B).

We also repeated all main analyses when deriving the spatial extent using alternative regional thresholds based on 2 SD from CU Aβ- participants (see Supplementary Results and Supplementary Figs. 10–14). Briefly, analyses related to memory and executive function remained similar. The notable difference was that the group of A-T+ significantly increased in CU and MCI, as tau thresholds, mostly in regions outside of the temporal lobe, were lowered.

## Discussion

We found that tau accumulation in late-onset sporadic Alzheimer’s disease, tau pathology and follows broad stages of pathological progression (i.e. Braak stages) uniformly across individuals, with early accumulation largely constrained to temporal lobe regions. However, abnormality in cortical tau at a finer-grain regional level is heterogeneous between participants, particularly as clinical symptoms progress. This effect was strongest in participants with mild cognitive impairment, who also showed the fastest region-to-region accumulation of abnormal tau across the whole brain. Finally, we also found that the spatial extent index was more strongly associated with executive function performance than temporal meta-ROI SUVR in participants with MCI or Alzheimer’s disease dementia and performed on par with temporal meta-ROI SUVR in other cognitive domains. This could be due in part to the topography of the associations between executive functions and tau burden which largely spans regions outside of the temporal lobe.

In line with the literature,^6^ we found that tau pathology usually accumulates in the entorhinal cortex (Braak I) before accumulating in other temporal regions (Braak III-IV)^7,27,32-34^ and finally large frontal and parietal regions (Braak V-VI).^8^ Similarly to previous work,^7,27,34,35^ this accumulation of abnormal amounts of tau pathology was mostly restricted to participants with high levels of Aβ—as opposed to Aβ-negative participants who showed little tau abnormality. An addition to our study is that these stages are followed not just cross-sectionally, but also over time. Overall, our results recapitulate and solidify our current understanding that tau pathology largely accumulates following the broad Braak stages in late-onset sporadic Alzheimer’s disease.

Despite these uniform broad inter-individual patterns, we found that within Braak stages, tau abnormality is regionally and inter-individually heterogeneous, especially in more advanced disease stages (i.e. MCI or Alzheimer’s disease). Alzheimer’s disease is known to present many different clinical variants^36^ and heterogeneous neuroimaging profiles.^15,37^ Specifically looking at tau pathology, several ‘subtypes’ of tau pathology have been suggested^13^ and different clinical variants of AD have also shown distinct tau deposition patterns.^14,16^ Other studies have used continuous variables of heterogeneity rather than subtypes, but always aggregating large swaths of brain regions together in smaller samples and with limited insight in more advanced participants.^38,39^ Furthermore, using individualized tau measures has been shown to better associate with future accumulation of tau pathology compared to using only Braak stages, demonstrating substantial inter-individual variability.^12^ As such, it is possible that while a large portion of the cortex may become abnormal following a specific sequence, regional patterns may differ between individuals. This was also suggested by a recent study which highlighted that despite tau pathology accumulating mostly in the temporal lobe, individualized regions of interest better capture change in tau over time.^17^ Our results suggest that this heterogeneity emerges in participants with MCI. Specifically, these participants accumulated abnormal amounts of tau pathology across the entire brain faster than CU participants and participants with Alzheimer’s disease, highlighting that the heterogenous accumulation of pathology appears once tau appears outside of the temporal lobe. On note, we also found that higher levels of tau pathology at baseline were associated with faster accumulation of tau pathology over time across diagnostic groups, but that the spatial extent seems to plateau at the stage of Alzheimer’s disease dementia. This suggests that there is a stage of the disease where the number of abnormal regions is reached, even though tangles (i.e. SUVR) continue to accumulate. This is somewhat contrary to Aβ pathology which seems to plateau over time at the late stage of the disease.^40^ Overall, these results suggest that fine-grain regional heterogeneity exists in tau deposition and accumulation, despite broad stages being followed uniformly, and that this heterogeneity starts to appear in participants with MCI.

Another key finding from the study is that the extent of tau pathology across the brain is associated with cognitive performance across cognitive domains on par with tau in the temporal meta-ROI in most domains, except for executive functioning where the spatial extent of tau was more strongly associated with cognition than the temporal meta-ROI. Literature in recent years has repetitively shown that tau—rather than Aβ—is the pathological hallmark most strongly associated with cognitive decline.^21^ This is also echoed by research on Alzheimer’s disease clinical variants. Previous work demonstrated that, while Aβ deposition patterns were similar across individuals from different clinical variants, tau patterns differ according to the variants, often affecting regions responsible for the main cognitive domain affected.^14,16^ This distinct topography of tau for each cognitive domain was also found in our study: tau was associated with the memory composite mostly in the temporal and frontal lobes bilaterally, tau was associated with the executive composite across the brain and tau was associated with language mostly unilaterally to the left hemisphere. Overall, our results suggest that regional tau topography is associated with specific cognitive domains, and that leveraging the spatial extent index may uncover stronger associations between tau and executive function performance.

### Strengths and limitations

The strengths of our study include a large sample size and a large longitudinal tau-PET sample. Cognition was collected over a long follow-up period; for at least 5 years in most cases.

Our study also has some limitations to acknowledge. We staged disease progression following the clinical diagnosis as attributed by physicians from memory clinics. However, not everyone with the same clinical label may be at the same ‘biological’ stage of the disease, i.e. two individuals with an MCI diagnosis may not have the same tau-PET patterns simply because they haven’t started to present symptoms at the same time.^13,41^ As such, the heterogeneity observed within each clinical diagnosis could be due to participants being at more advanced disease stages. Furthermore, we use the overlap of spatial extent patterns to define heterogeneity which somewhat lacks spatial resolution. It is possible that the same index, e.g. 0.5, represents the positive overlap of a small set of regions spatially close to one another or the overlap of a large set of regions spatially distant from one another. Nonetheless, our results are reassuring: if biological staging had been the driver of the heterogeneity in the tau patterns cross-sectionally, our longitudinal results would have shown that participants had less (not more) heterogeneity, and we replicated most of the findings of heterogeneity within each Braak stage.

Our main method to derive the spatial extent index relies on unsupervised GMM, where participants are clustered in either one of two groups: ‘normal’ or ‘abnormal’ tau. Some of the limitations of these methods include the need to set in advance certain components of the models, including the number of clusters—two in the case of this paper—which may preclude more complex underlying patterns in the data. However, we ensured that a two-component method was a better fit compared to a single component using the Bayesian information criterion. Due to the data-driven nature of the method, should the proportion of participants with high levels of tau included change, the thresholds will also likely change. However, in supplementary analyses, we showed that GMM thresholds were likely less influenced by outliers in the data compared to other methods for deriving thresholds such as 2 SD from CU Aβ− participants. This suggests that other traditional methods are likely plagued by the same issue, and the main results of the paper were not dependent on the choice of how to derive the thresholds.

A major limitation is ADNI’s inclusion criteria. By design, ADNI includes participants with amnestic disease presentation.^42^ However, atypical variants of AD may not present with memory impairment at the forefront of their cognitive complaints.^36^ As such, ADNI’s sample may be by design very homogenous. This could explain why the spatial extent performed relatively similarly to the meta-ROI across cognitive composites. Despite this homogenous sample, we still found heterogenous tau patterns and diverse tau-cognition associations, and a stronger association of individualized measures with executive functioning and language.

Many participants have the same visuospatial score cross-sectionally and over time, which could stem from an error in the database. Any results related to this composite index should be carefully considered in this context.

Finally, due to molecular limitations of the flortaucipir tracer which presents off-target binding in the hippocampus, we excluded this region from our spatial extent index. However, there is convincing evidence that some patients with Alzheimer’s disease dementia will present more (medial temporal subtype) or less (hippocampal sparring subtype) neurodegeneration in the hippocampus,^37^ suggesting that tau within the hippocampus is also an important source of heterogeneity which is missed by the spatial extent index from the current paper. Future research should aim to confirm these findings using tau-PET tracers less sensitive to off-target binding in the hippocampus such as MK6240.

## Conclusion

While our study confirms that participants accumulate tau pathology following the broad Braak stages, we also demonstrate that regional accumulation is subject to significant heterogeneity—particularly as the disease progresses. This heterogeneity seems to take hold during the MCI stage, as these participants accrue more tau abnormal regions faster than both CU and participants with Alzheimer’s disease dementia. We also illustrate that the topography of the tau pathology is differentially associated with cognitive domains, and that using the spatial extent (i.e. tau abnormality across the brain) can lead to stronger associations with executive functioning. Taken together, our results suggest that using regional tau is important, particularly when considering participants with MCI or Alzheimer’s disease dementia, and we propose a simple research method to investigate these regionalities going forward, perhaps in the context of atypical Alzheimer’s disease.

## Supplementary material

Supplementary material is available at *Brain Communications* online.

## Supplementary Material

fcae031_Supplementary_Data

## Data Availability

Data used in this study come from the ADNI. Investigators interested in obtaining the data can apply for access on ADNI’s website: https://adni.loni.usc.edu/. The code used to compute the spatial extent measures is publicly available in *sihnpy* as of version v0.2, a Python package freely available for download (https://sihnpy.readthedocs.io/). The code used for the statistical analyses and the figures is also made available freely on GitHub (https://github.com/villeneuvelab/projects).
