# Supplementary material for: Tau accumulation and its spatial progression across the Alzheimer’s disease spectrum
Source: Brain Commun. 2024 Feb 7;6(1):fcae031. doi: 10.1093/braincomms/fcae031 (PMC10896475; doi:10.1093/braincomms/fcae031)
Supplement: fcae031_Supplementary_Data [file fcae031_supplementary_data.pdf]

Tau accumulation and its spatial progression across the Alzheimer's disease spectrum

Supplementary material

<u>**Table of contents**</u>

Supplementary Results p.3

Supplementary Figure 1 Spatial extent of abnormal tau deposition and accumulation in amyloid negative participants of the ADNI cohort p.5

Supplementary Figure 2 Tau measures by amyloid and clinical status p.7

Supplementary Figure 3 Annual change in tau-PET measures p.8

Supplementary Figure 4 Heterogeneity in tau-PET spatial extent at baseline and longitudinally p.9

Supplementary Figure 5 Association between tau-PET measures, and executive functioning performance and decline p.10

Supplementary Figure 6 Association between tau-PET measures, and language performance and decline p.12

Supplementary Figure 7 Association between tau-PET measures, and visuospatial performance and decline p.14

Supplementary Figure 8 Association between tau measures and age p.16

Supplementary Figure 9 Amyloid and tau status in the cohort using thresholds from cognitively unimpaired Aβ- participants p.17

Supplementary Figure 10 Spatial extent using CU-based thresholds abnormal tau deposition in amyloid-positive participants of the ADNI cohort p.18

Supplementary Figure 11 Association between spatial extent index derived from CU Aβ-thresholds, and cognitive performance p.19

Supplementary Figure 12 Association between spatial extent index derived from CU Aβ-thresholds, and cognitive decline over time p.20

Supplementary Figure 13 Comparison of spatial extent approaches p.21

Supplementary Figure 14 Spatial extent of tau abnormality in CU Aβ+ participants at baseline p.22

Supplementary Table 1 Regional thresholds of tau positivity based on Gaussian Mixture Modelling p.23

Supplementary Table 2 Regional thresholds of tau positivity based on 2 standard deviation from cognitively unimpaired Aβ- participants p.24

Supplementary Table 3 Regional tau abnormality across regions of interest p.25

Supplementary Table 4 Regional tau abnormality progression across regions of interest p.26

Supplementary Table 5 Braak stages thresholds p.27

Supplementary results

We replicated our main results using a spatial extent index derived from regional thresholds using 2 standard deviation from the mean of CU Aβ- participants instead of the GMM approach. Using the CU-based thresholds, we recharacterized AT status in the cohort (Supplementary Figure 9; original Figure 2A). The proportion of A+T+ (12.1% to 18.4%; $\chi^2 = 27.034$, $p < 0.001$) and A-T+ (3.2% to 14.9%; $\chi^2 = 50.019$, $p < 0.001$) increased in CU participants with the CU-based thresholds. We found similar results in participants with MCI for the proportion of A+T+ (36.1% to 41.1%; $\chi^2 = 13.067$, $p < 0.001$) and the proportion of A-T+ participants (8.7% to 16.9%; $\chi^2 = 21.043$, $p < 0.001$). The proportions did not change in participants with AD. Additionally, the proportion of A-T+ participants increased more than the proportion of A+T+ participants in CU participants ($\chi^2 = 5.975$, $p = 0.015$), but not in participants with MCI ($\chi^2 = 1.290$, $p = 0.256$). Recreating the heatmap represented in Figure 3A using the CU-based thresholds (Supplementary Figure 10), we found that our previous main findings remained: Braak stages were followed across all Braak stages up to and including Braak V in over 84% of participants, while patterns were heterogenous within regions.

We tested whether spatial extent index derived from CU Aβ- thresholds would retain its advantage over the temporal meta-ROI SUVR in cognitive performance (Supplementary Figure 12) and decline (Supplementary Figure 13). Overall, the CU-derived spatial extent index was associated similarly with cognition than the GMM-based measure across cognitive domains. However, the CU-derived spatial extent index was no longer superior to the temporal meta-ROI SUVR in the association with executive performance at baseline in participants with MCI ($z = -0.742$, $p = 0.771$) or AD ($z = -1.534$, $p = 0.063$) and over time for participants with MCI ($z = -0.007$, $p = 0.497$). It was also no longer superior for the language performance at baseline in participants with MCI ($z = -0.983$, $p = 0.163$). Interestingly, in some language and visuospatial associations, less cognitive decline was associated with more CU-based spatial extent index.

We investigated what could be driving the differences between both methods. First, we found that, on average, thresholds from the GMM were 0.10 SUVR ($t = 20.57$, $df = 69$, $p < 0.001$) higher than thresholds from CU Aβ- participants, but this varied across the brain where left temporal regions presented the least difference between methods and frontal regions presenting the most

(Supplementary Figure 9A). Second, we investigated the extent to which the distribution of tau in CU Aβ- participants influenced the thresholds derived from both methods. We considered the distribution of tau using the max-min range (spread of the entire data). Considering the total range of each region, spatial extent from the GMM ($r = 0.31$, $p = 0.009$; Supplementary Figure 9C) was associated with the range, but the spatial extent from the 2SD CU Aβ- method showed a stronger association with the total range ($r = 0.66$, $p < 0.001$; Supplementary Figure 9D), which was higher than the spatial extent from the GMM ($z = -5.43$, $p < 0.001$).

Supplementary Figure 1

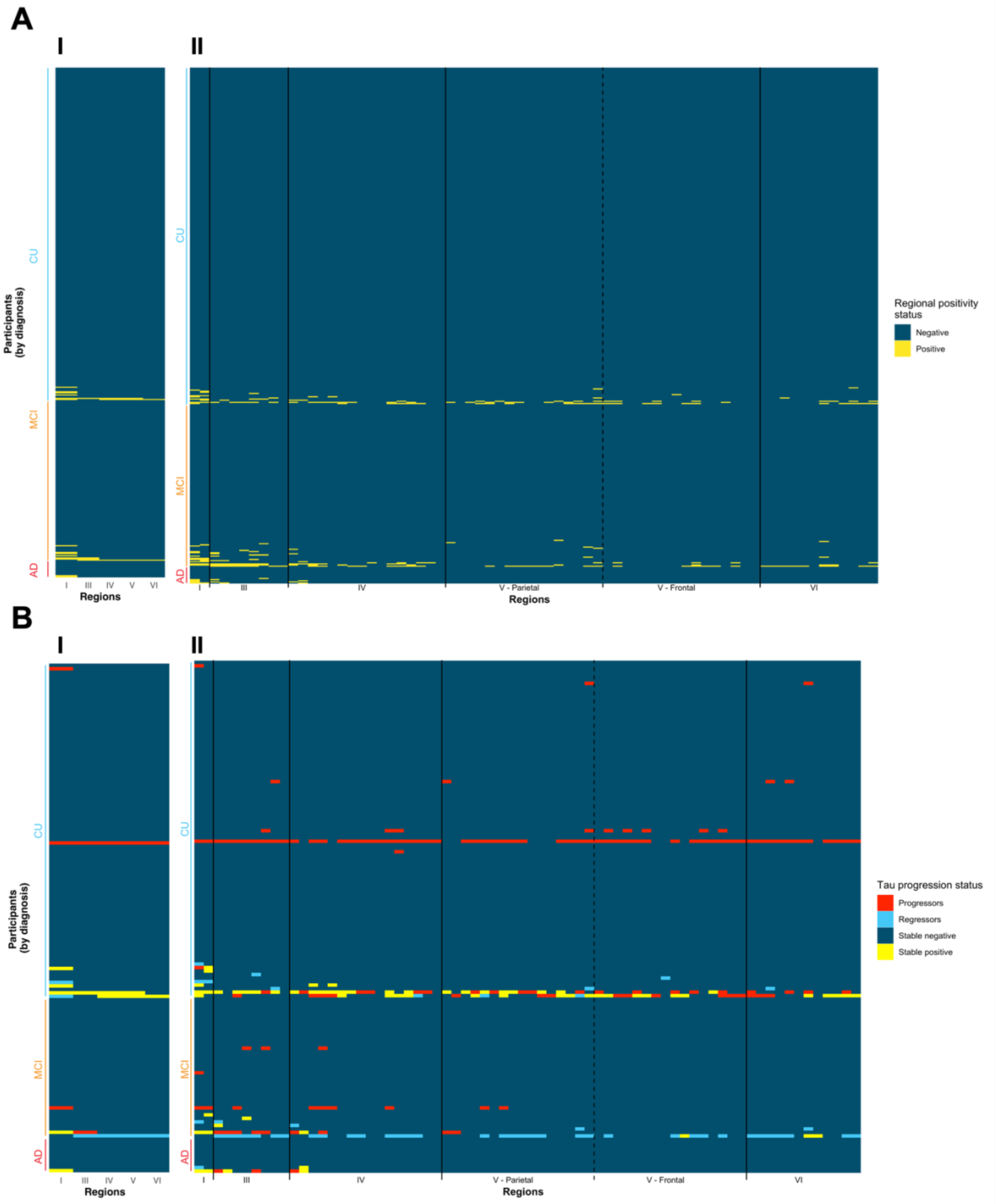

Supplementary Figure 1 Spatial extent of abnormal tau deposition and accumulation in amyloid negative participants of the ADNI cohort. (**A**) Based on the method discussed in Figure

1, abnormality thresholds were determined for each (**I.**) Braak stages (except stage II) and for each (**II.**) region of the cortical mantle and the bilateral amygdalae (70 regions). One row on the heatmap correspond to an individual participant, while each column represents a distinct cortical region. Within each diagnostic group, participants were sorted from individuals with lowest to highest spatial extent index. Regions on the x-axis in **II.** are sorted by Braak stages. (**B**) Abnormal accumulation is presented by (**I.**) Braak stages and (**II.**) all 70 individual brain regions of the Desikan atlas. Colors denote the change in the region between the baseline and the last available visit. A stable region (negative or positive; blue or yellow) did not change status during the follow-up. A progressing region (red) was originally negative and subsequently became positive over time. A regressing region (teal) was originally positive and became negative over time.

Supplementary Figure 2

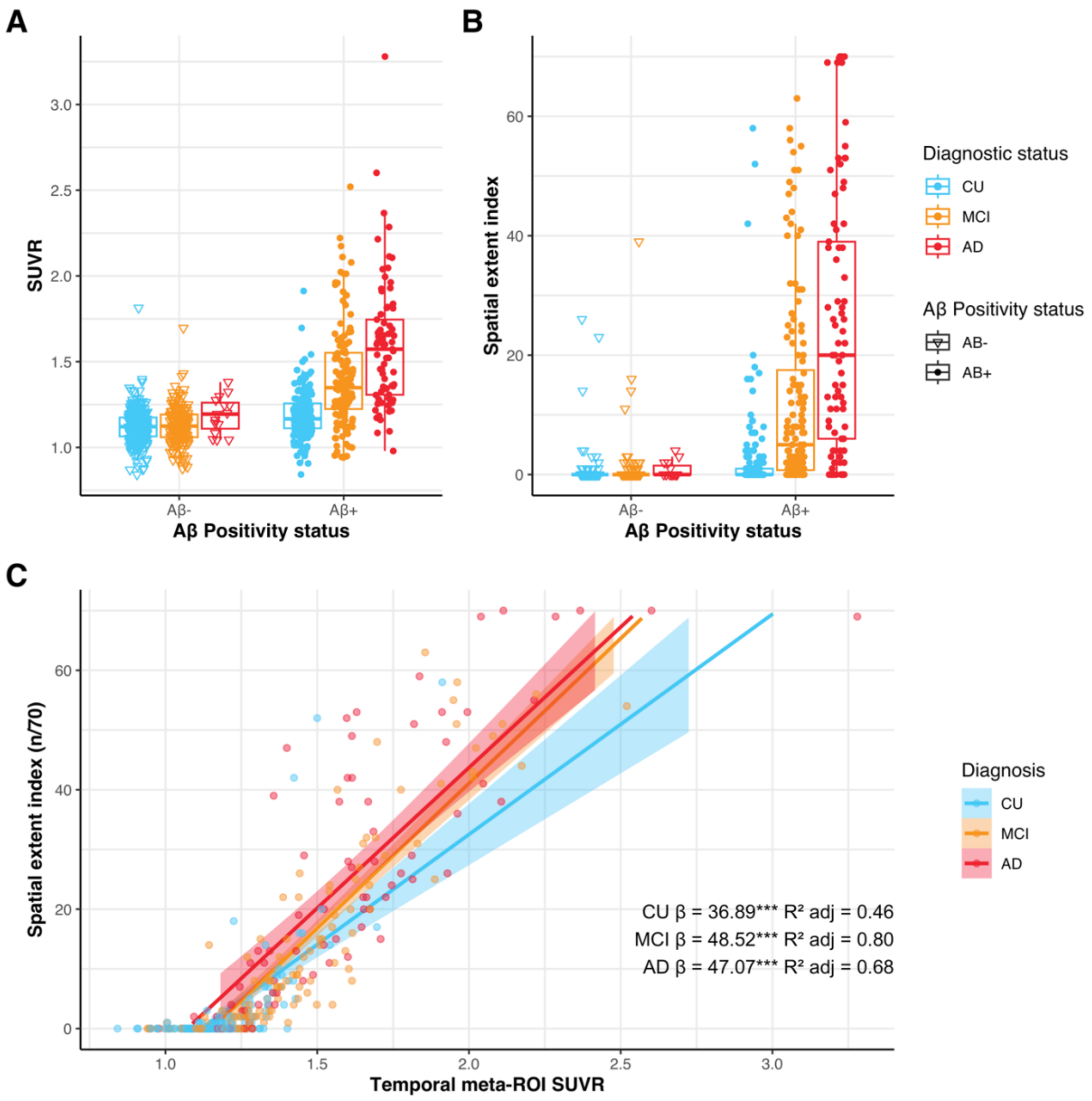

Supplementary Figure 2 Tau measures by amyloid and clinical status. (A) Average tau SUVR within the temporal meta-ROI by amyloid positivity and diagnostic status. (**B**) Spatial extent index (i.e., number of tau abnormal regions) by amyloid positivity and diagnostic status. In both panels, ANOVAs were used to compare tau measures between Aβ- and Aβ+ participants (e.g., Aβ- compared to Aβ+ cognitively unimpaired participants). As all analyses yielded that Aβ+ had more tau—across all diagnostic groups—at p < 0.001 significance, we did not plot the model significance on the figure. (**C**) Association using linear models between temporal meta-ROI SUVR and spatial extent index in Aβ+ participants of each diagnostic group. Beta coefficients and adjusted R^2 are presented at the bottom of the graph (*** = P < 0.001).

Supplementary Figure 3

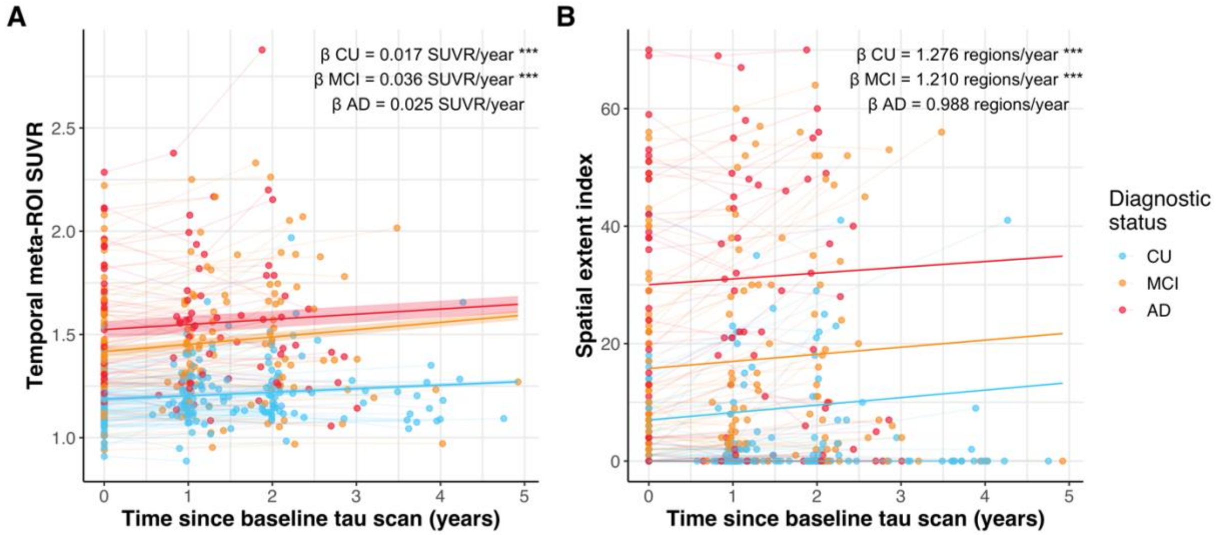

Supplementary Figure 3 Annual change in tau-PET measures. Annual change of (A) standardized uptake value ratio (SUVR) in the temporal meta-ROI and annual change of (B) spatial extent index in CU (blue), MCI (orange) and AD (red). Rates of annual change, computed with linear mixed models for temporal meta-ROI SUVR and zero-inflated Poisson mixed models for the spatial extent index, are presented at the top right corner of the graphs. Models' significance when controlling for age, sex and education are denoted by stars next to the rate. Brackets and stars between two slopes denote a significant group difference in the rate of change (* = P < 0.05).

Supplementary Figure 4

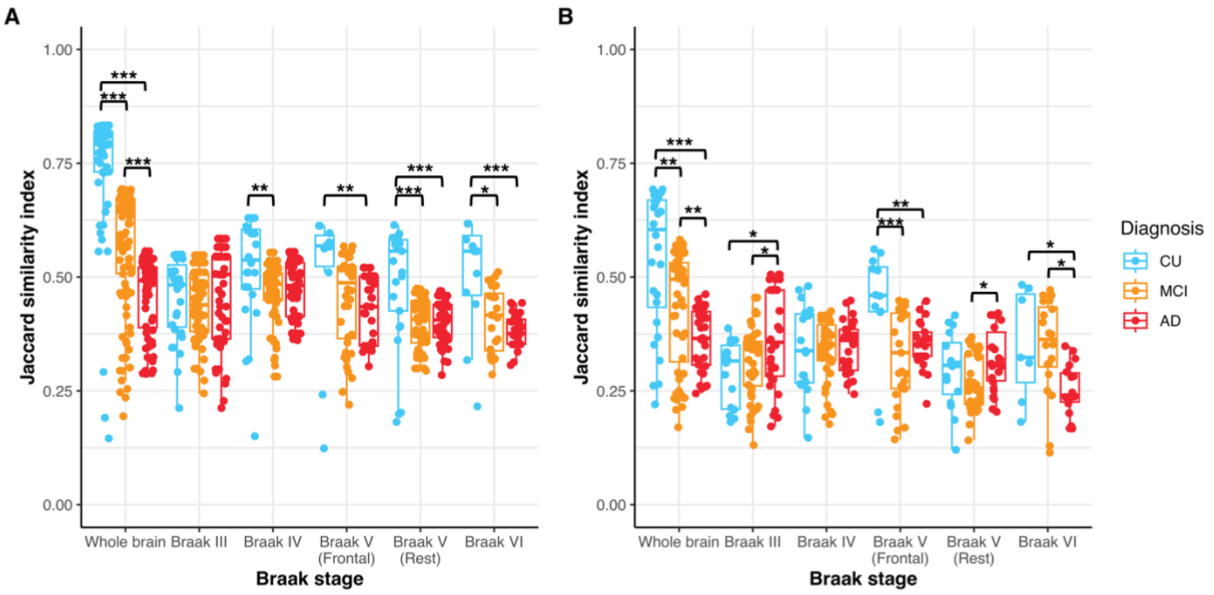

Supplementary Figure 4 Heterogeneity in tau-PET spatial extent at baseline and longitudinally. (**A**) Across the whole brain and within each Braak stage, we computed how, on average, the patterns of tau abnormality (i.e., positivity for specific sets of brain regions) overlapped between participants of the same diagnostic group using the Jaccard similarity index. An index closer to one means a bigger overlap on average between participants in terms of regions that are positive, while an index closer to zero means more heterogeneity on average between participants. For each diagnostic group, we only retained participants who had at least one tau positive region. (**B**). Across the brain we computed how, on average, the patterns of change in tau abnormality (i.e., stability, progression, or regression for specific sets of brain regions) overlapped between participants of the same diagnostic group using the same method described in (**A**)**.** In both (**A**) and (**B**), difference in average similarity was compared using Kruskal-Wallis tests. Post-hoc Dunn tests (with Bonferroni correction) were conducted when the result was significant. * = $P < 0.05$, ** = $P < 0.01$, *** = $P < 0.001$. CU = Cognitively unimpaired, MCI = Mild cognitive impairment, AD = Alzheimer's disease

Supplementary Figure 5

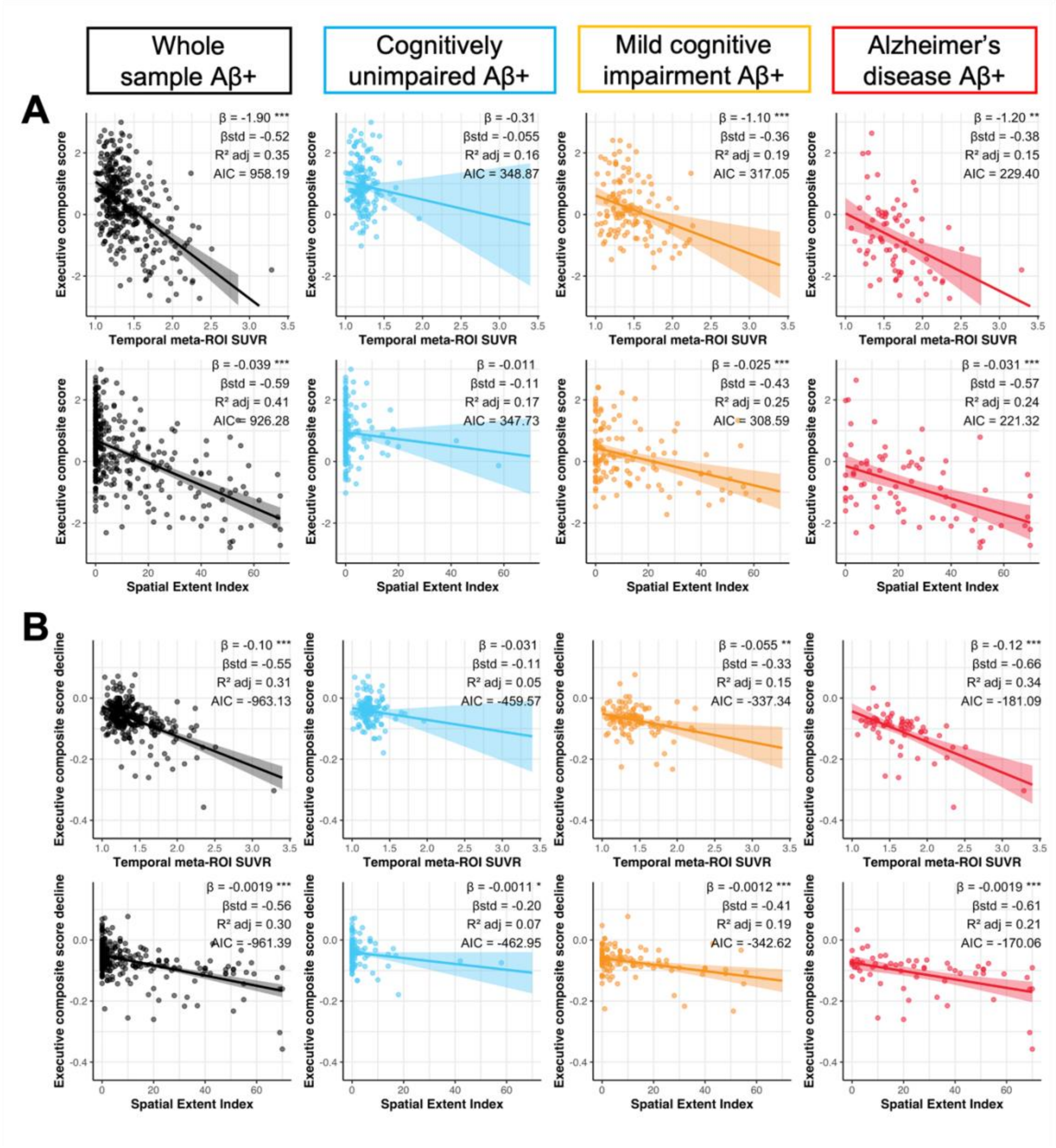

Supplementary Figure 5 Association between tau-PET measures, and executive functioning performance and decline. (**A**) Executive functioning performance closest in time to the tau-PET scan and (**B**) executive functioning decline computed across the study period were associated to both temporal meta-ROI SUVR and spatial extent index in Aβ+ participants using linear regressions. Cognitive decline was computed for each participant with more than two cognitive timepoints using linear mixed effect models with random slopes and intercepts. In each panel, columns represent a diagnostic group (leftmost/black: whole sample, second from the left/blue: cognitively unimpaired, second from the right/orange: mild cognitive impairment, right-most/red: Alzheimer's disease). Simple and standardized β coefficients, *adjusted* R^2 and *AIC*, controlled for

age sex and education, are shown on the graphs. *P*-value of models are indicated next to the simple beta coefficients. (° : $P < 0.1$, * : $P < 0.05$, ** : $P < 0.01$, *** $P < 0.001$) Results remained significant after a multiple comparison false discovery rate (FDR) correction.

Supplementary Figure 6

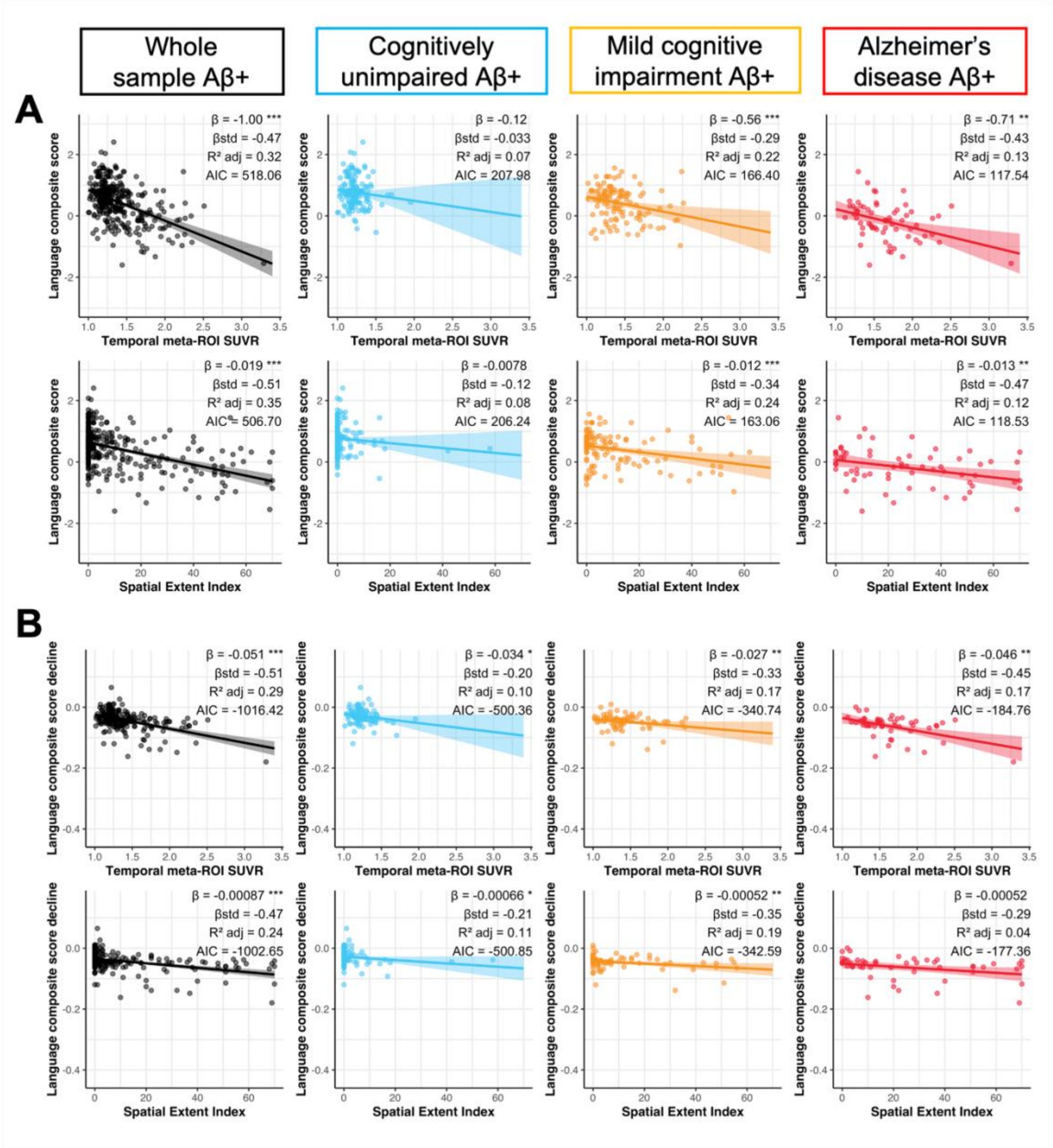

Supplementary Figure 6 Association between tau-PET measures, and language performance and decline. (**A**) Language performance closest in time to the tau-PET scan and (**B**) language decline computed across the study period were associated to both temporal meta-ROI SUVR and spatial extent index in Aβ+ participants using linear regressions. Cognitive decline was computed for each participant with more than two cognitive timepoints using linear mixed effect models with random slopes and intercepts. In each panel, columns represent a diagnostic group (leftmost/black: whole sample, second from the left/blue: cognitively unimpaired, second from the right/orange: mild cognitive impairment, right-most/red: Alzheimer's disease). Simple and standardized β coefficients, *adjusted* R^2 and *AIC*, controlled for age sex and education, are shown on the graphs.

P-value of models are indicated next to the simple beta coefficients. (° : $P < 0.1$, * : $P < 0.05$, ** : $P < 0.01$, *** $P < 0.001$) Results remained significant after a multiple comparison false discovery rate (FDR) correction.

Supplementary Figure 7

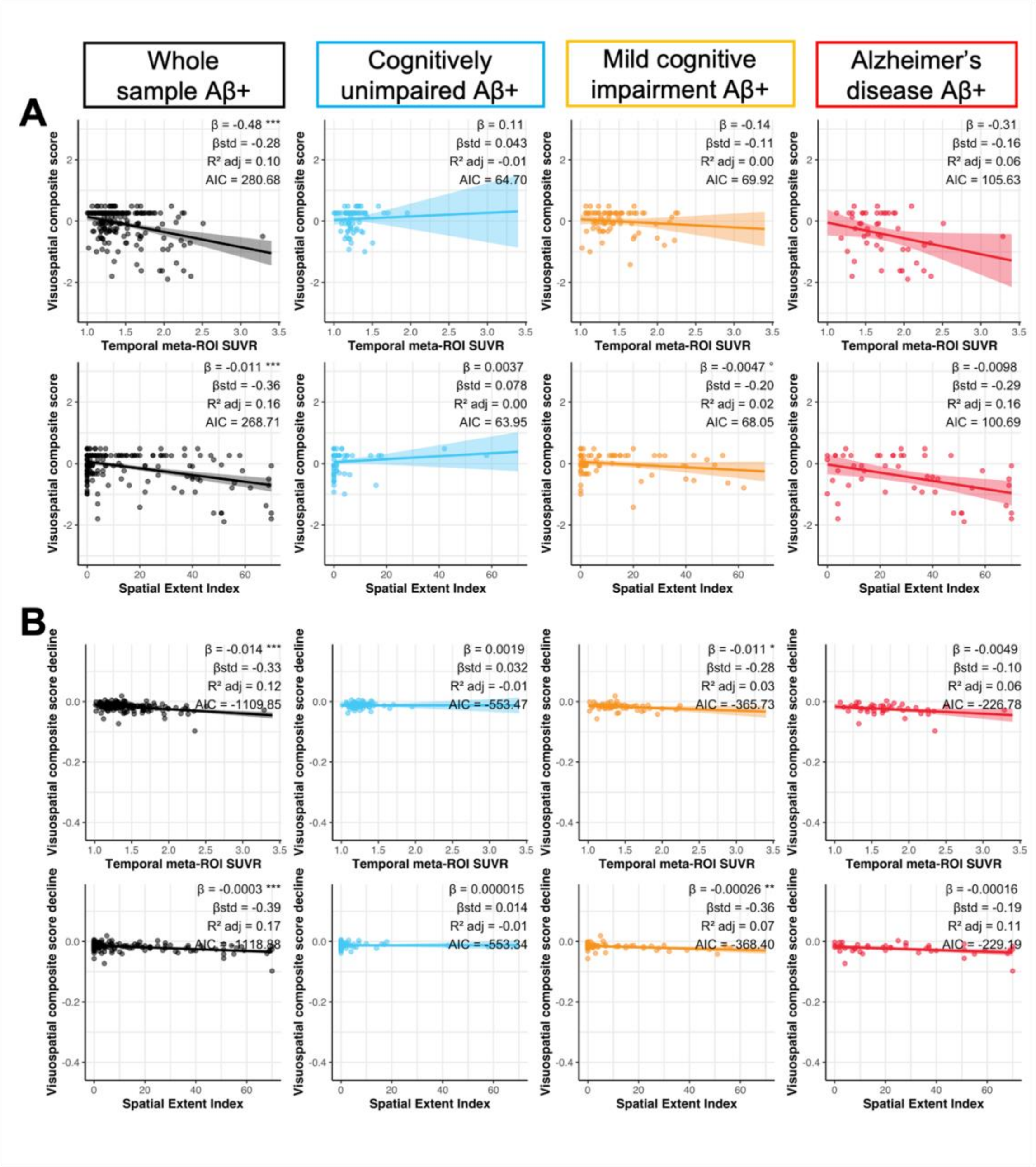

Supplementary Figure 7 Association between tau-PET measures, and visuospatial performance and decline. (**A**) Visuospatial performance closest in time to the tau-PET scan and (**B**) visuospatial decline computed across the study period were associated to both temporal meta-ROI SUVR and spatial extent index in Aβ+ participants using linear regressions. Cognitive decline was computed for each participant with more than two cognitive timepoints using linear mixed effect models with random slopes and intercepts. In each panel, columns represent a diagnostic group (leftmost/black: whole sample, second from the left/blue: cognitively unimpaired, second from the right/orange: mild cognitive impairment, right-most/red: Alzheimer's disease). Simple

and standardized β coefficients, *adjusted* R^2 and *AIC*, controlled for age sex and education, are shown on the graphs. *P*-value of models are indicated next to the simple beta coefficients. (° : $P < 0.1$, * : $P < 0.05$, ** : $P < 0.01$, *** $P < 0.001$) Results remained significant after a multiple comparison false discovery rate (FDR) correction.

Supplementary Figure 8

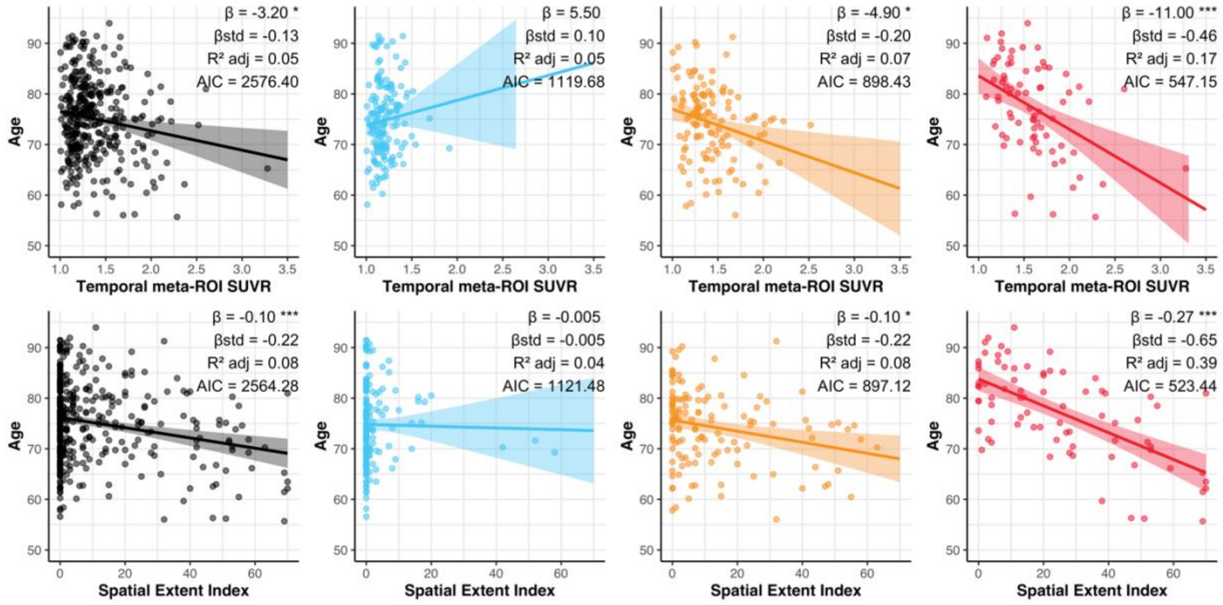

Supplementary Figure 8 Association between tau measures and age. Age at baseline was associated with temporal meta-ROI SUVR (first row) and spatial extent index (second row) in Aβ+ participants using linear regressions. In each panel, columns represent a diagnostic group (leftmost/black: whole sample, second from the left/blue: cognitively unimpaired, second from the right/orange: mild cognitive impairment, right-most/red: Alzheimer's disease). Simple and standardized β coefficients, adjusted R^2 and AIC, controlled for sex and education, are shown on the graphs. P-values of models are indicated next to the simple beta coefficients. (° : P < 0.1, * : P < 0.05, ** : P < 0.01, *** P < 0.001) Results remained significant after a multiple comparison false discovery rate (FDR) correction.

Supplementary Figure 9

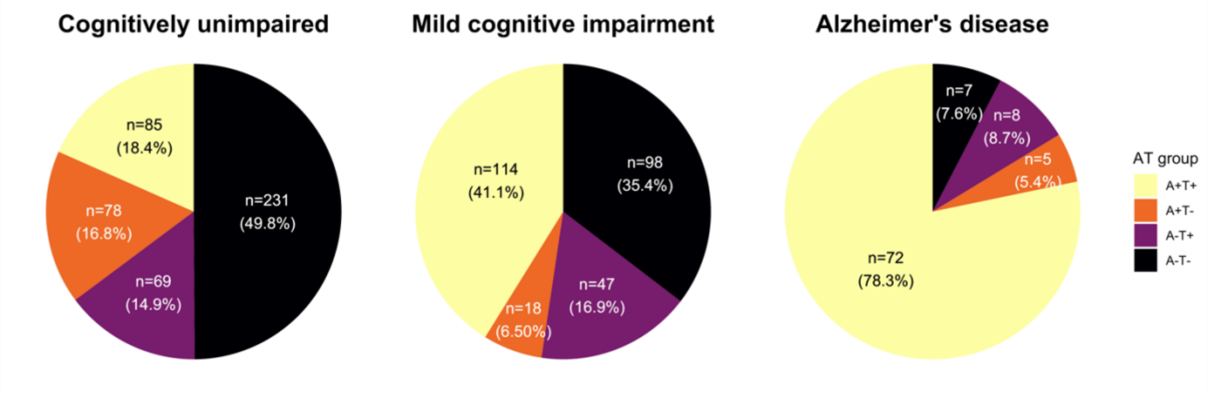

Supplementary Figure 9 Amyloid and tau status in the cohort using thresholds from cognitively unimpaired Aβ- participants. Aβ positivity was established using ADNI's tracer-specific recommendations for both Florbetapir and Florbetaben. Tau positivity was defined as having at least one region positive for tau pathology (spatial extent index of one and above). The spatial extent was derived across regions by using the mean plus two standard deviations of tau-PET values in CU Aβ- participants.

Supplementary Figure 10

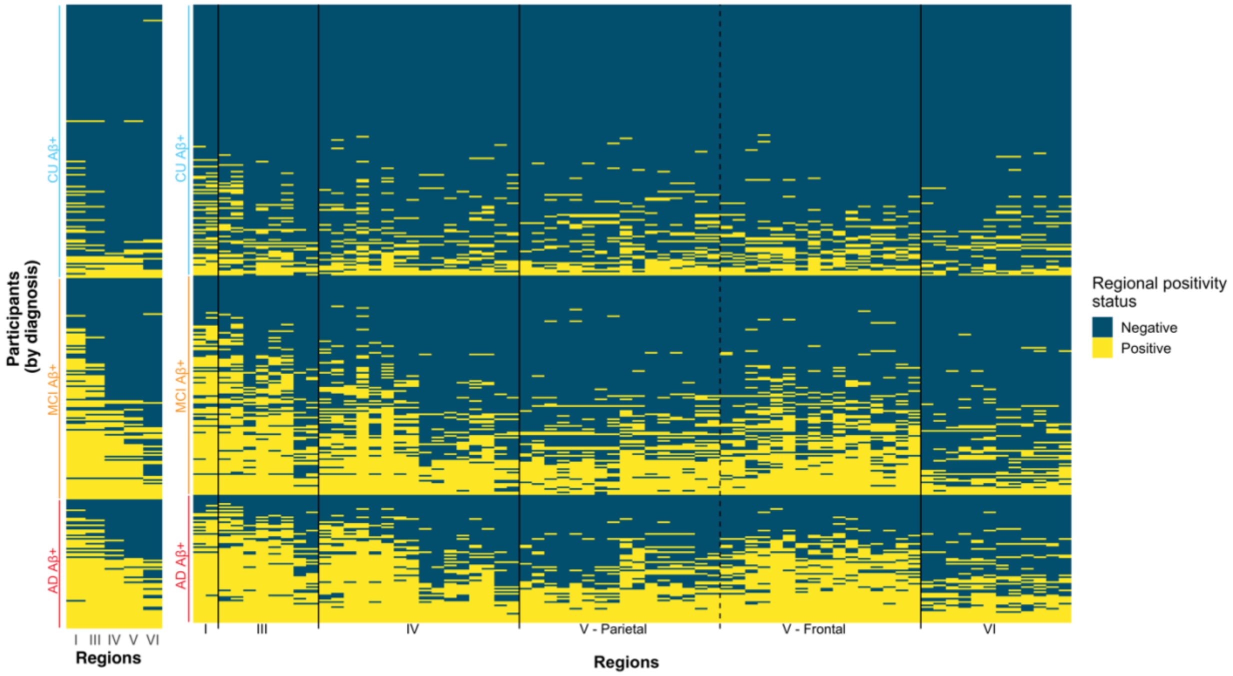

Supplementary Figure 10 Spatial extent using CU-based thresholds abnormal tau deposition in amyloid-positive participants of the ADNI cohort. Replication of Figure 3A, but the regional spatial extent was instead derived from thresholds based on the mean plus two standard deviations of CU Aβ- participants. Abnormality thresholds were determined for each **I.** Braak stages (except stage II) and for each **II.** region of the cortical mantle and the bilateral amygdalae (70 regions). One row on the heatmap correspond to an individual participant, while each column represents a distinct cortical region. Within each diagnostic group, participants were sorted from individuals with lowest to highest spatial extent index. Regions on the x-axis in **II.** are sorted by Braak stages.

Supplementary Figure 11

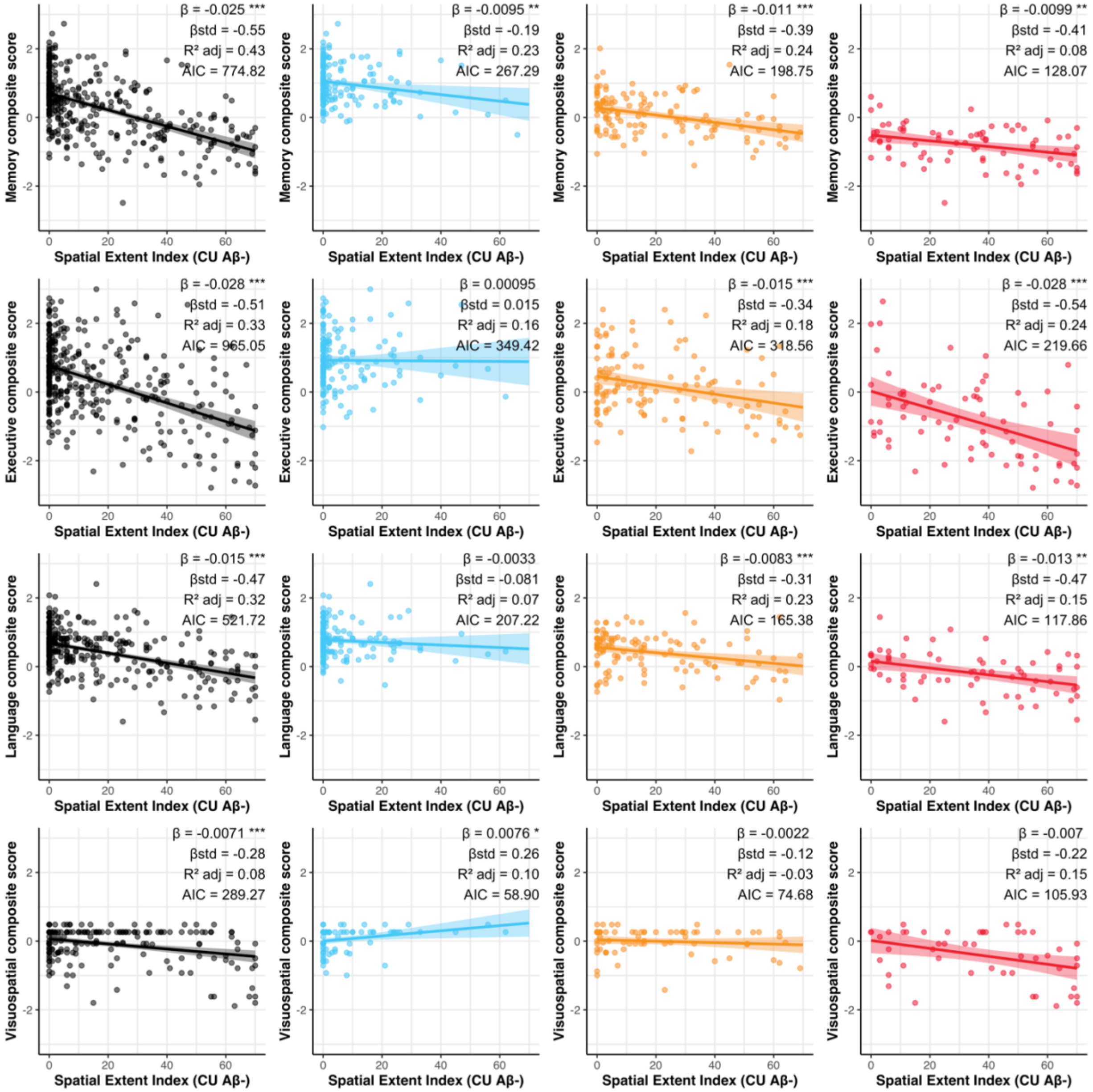

Supplementary Figure 11 Association between spatial extent index derived from CU Aβ- thresholds, and cognitive performance. Association between the cognitive performance closest in time to the tau-PET scan and cognitive performance at baseline. Each row represents a cognitive measure (from top to bottom, memory, executive function, language and visuospatial) and each column represents a diagnostic group (leftmost/black: whole sample, (leftmost/black: whole sample, second from the left/blue: cognitively unimpaired, second from the right/orange: mild cognitive impairment, right-most/red: Alzheimer's disease). Simple and standardized β coefficients, adjusted R^2 and AIC, controlled for age sex and education, are shown on the graphs. P-value of models are indicated next to the simple beta coefficients. (° : P < 0.1, * : P < 0.05, ** : P < 0.01, *** P < 0.001) Results remained significant after a multiple comparison false discovery rate (FDR) correction.

Supplementary Figure 12

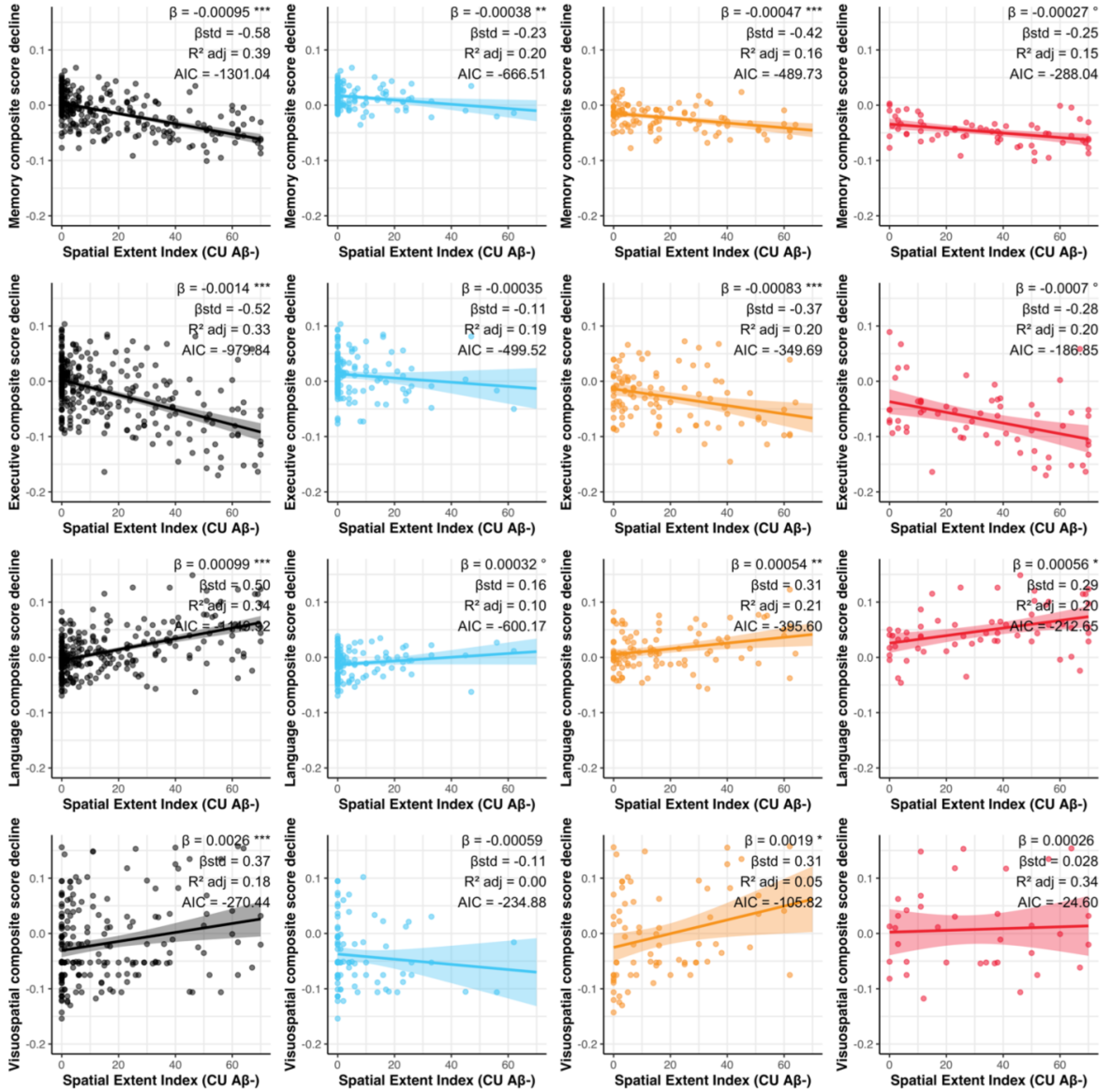

Supplementary Figure 12 Association between spatial extent index derived from CU Aβ- thresholds, and cognitive decline over time. Association between the cognitive performance closest in time to the tau-PET scan and cognitive decline over time. For each domain, cognitive decline was computed for each participant with more than two cognitive timepoints using linear mixed-effect models with random slopes and intercepts. Each row represents a cognitive domain (from top to bottom, memory, executive function, language and visuospatial) and each column represents a diagnostic group (leftmost/black: whole sample, (leftmost/black: whole sample, second from the left/blue: cognitively unimpaired, second from the right/orange: mild cognitive impairment, right-most/red: Alzheimer's disease). Simple and standardized β coefficients, adjusted R^2 and AIC, controlled for age sex and education, are shown on the graphs. P-value of models are indicated next to the simple beta coefficients. (° : $P < 0.1$, * : $P < 0.05$, ** : $P < 0.01$, *** $P < 0.001$) Results remained significant after a multiple comparison false discovery rate (FDR) correction.

Supplementary Figure 13

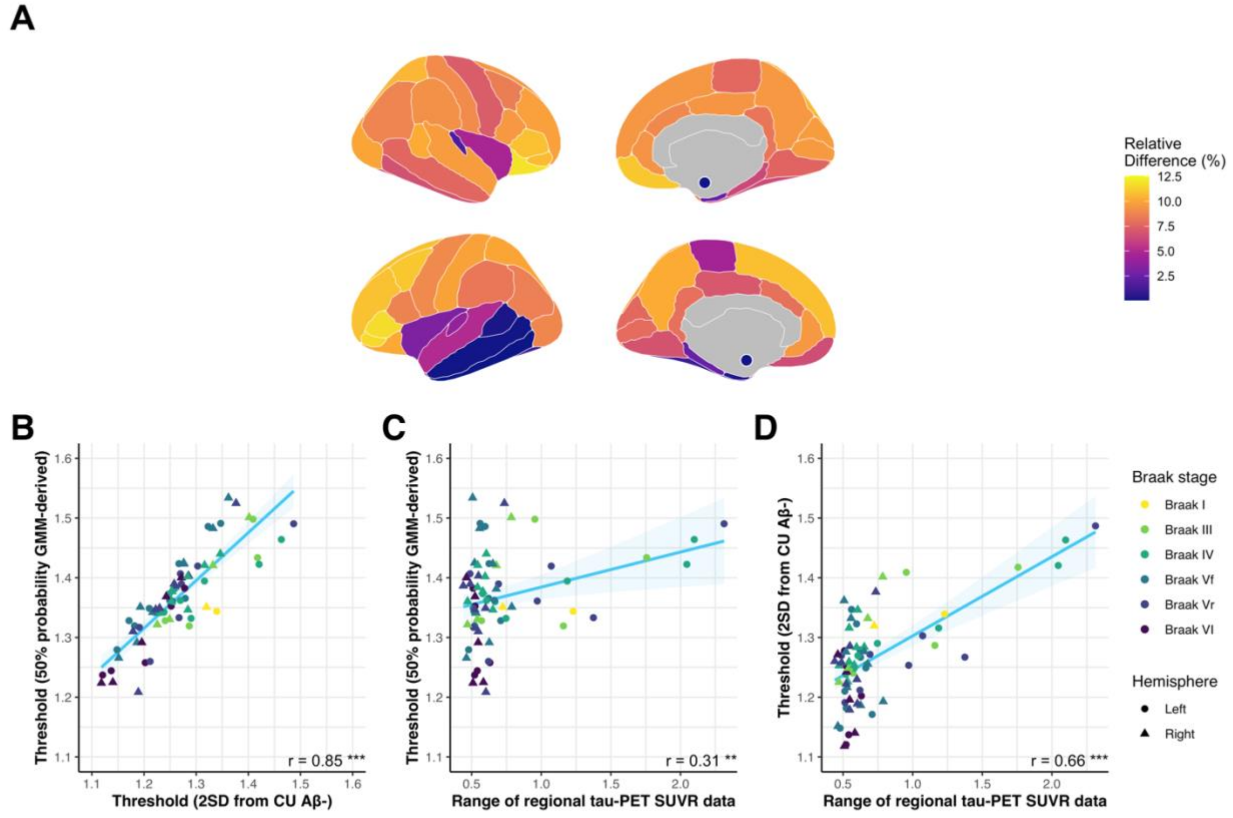

Supplementary Figure 13 Comparison of spatial extent approaches. A) The percent difference in thresholds for tau-PET positivity across brain regions derived from applying GMM on the whole ADNI sample or by taking 2SD from the mean of CU Aβ-. On average, thresholds from the GMM were 0.10 SUVR higher than the 2SD method. The small sphere in the medial view of the brain represents the amygdala. B) Correlation between thresholds from the GMM method and 2SD from the mean of CU Aβ-. C) Correlation between the regional thresholds derived from the GMM and the range (max-min) of SUVR values in all regions. D) Correlation between the regional thresholds derived from the 2SD CU Aβ- method and the range (min-max) of SUVR values in all regions. In all three panels, each point corresponds to a brain region, with the colour representing their Braak stage and the shape the left or the right hemisphere (** : P < 0.01, *** P < 0.001).

Supplementary Figure 14

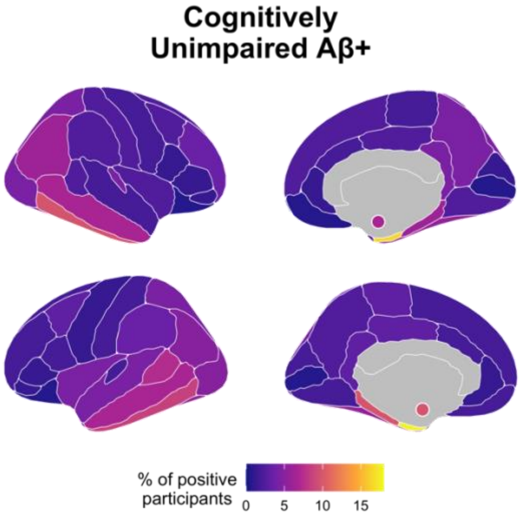

Supplementary Figure 14 Spatial extent of tau abnormality in CU Aβ+ participants at baseline. Replication of Figure 3C for CU Aβ+ participants, but with a colour scale allowing to see the pattern specific to this group.

Supplementary Table 1 Regional thresholds of tau positivity based on Gaussian Mixture Modelling

Braak stage	Region	Threshold value (SUVR) LH	RH
I	entorhinal	1.344	1.349
III	amygdala	1.498	1.501
	fusiform	1.434	1.421
	parahippocampal	1.319	1.330
	lingual	1.328	1.322
IV	temporalpole	1.395	1.402
	inferiortemporal	1.464	1.440
	middletemporal	1.423	1.421
	isthmuscingulate	1.360	1.360
	caudalanteriorcingulate	1.342	1.372
	insula	1.332	1.346
	posteriorcingulate	1.362	1.384
	rostralanteriorcingulate	1.414	1.391
V_1	lateraloccipital	1.390	1.390
	inferiorparietal	1.420	1.403
	superiortemporal	1.333	1.347
	bankssts	1.490	1.525
	precuneus	1.407	1.387
	superiorparietal	1.317	1.310
	supramarginal	1.361	1.349
	transversetemporal	1.260	1.209
V_2	parsopercularis	1.362	1.381
	parsorbitalis	1.486	1.483
	parstriangularis	1.424	1.423
	frontalpole	1.328	1.357
	caudalmiddlefrontal	1.320	1.292
	lateralorbitofrontal	1.491	1.534
	medialorbitofrontal	1.462	1.425
	rostralmiddlefrontal	1.349	1.342
	superiorfrontal	1.280	1.266
VI	pericalcarine	1.382	1.400
	cuneus	1.353	1.369
	paracentral	1.258	1.292
	postcentral	1.237	1.229
	precentral	1.244	1.225

LH = Left hemisphere, RH = Right hemisphere, SUVR = Standardized uptake value ratio. V_2 represents Braak V region in the frontal lobe while V_1 regroups the remaining Braak V regions.

Supplementary Table 2 Regional thresholds of tau positivity based on 2 standard deviation from cognitively unimpaired Aβ- participants

Braak stage	Region	Threshold value (SUVR) LH	RH
I	entorhinal	1.339	1.320
III	amygdala	1.409	1.402
	fusiform	1.418	1.332
	parahippocampal	1.287	1.249
	lingual	1.240	1.225
IV	temporalpole	1.316	1.284
	inferiortemporal	1.463	1.346
	middletemporal	1.421	1.316
	isthmuscingulate	1.255	1.253
	caudalanteriorcingulate	1.238	1.226
	insula	1.290	1.284
	posteriorcingulate	1.270	1.259
	rostralanteriorcingulate	1.249	1.254
V_1	lateraloccipital	1.249	1.256
	inferiorparietal	1.323	1.332
	superiortemporal	1.267	1.277
	bankssts	1.171	1.193
	precuneus	1.182	1.186
	superiorparietal	1.347	1.362
	supramarginal	1.279	1.276
	transversetemporal	1.210	1.217
V_2	parsopercularis	1.148	1.151
	parsorbitalis	1.272	1.258
	parstriangularis	1.303	1.285
	frontalpole	1.267	1.222
	caudalmiddlefrontal	1.487	1.376
	lateralorbitofrontal	1.270	1.260
	medialorbitofrontal	1.191	1.179
	rostralmiddlefrontal	1.253	1.230
	superiorfrontal	1.212	1.189
VI	pericalcarine	1.278	1.272
	cuneus	1.252	1.243
	paracentral	1.202	1.196
	postcentral	1.121	1.118
	precentral	1.137	1.140

LH = Left hemisphere, RH = Right hemisphere, SUVR = Standardized uptake value ratio. V_2 represents Braak V region in the frontal lobe while V_1 regroups the remaining Braak V regions.

Supplementary Table 3 Regional tau abnormality across regions of interest

Braak stage	Region	CU Aβ+ (n = 163) LH	RH	MCI Aβ+ (n = 132) LH	RH	AD Aβ+ (n = 77) LH	RH	CU Aβ- (n = 300) LH	RH	MCI Aβ- (n = 145) LH	RH	AD Aβ- (n = 15) LH	RH
I	entorhinal *	17.8	16.6	58.3	61.4	74	75.3	2	2	6.2	4.8	26.7	6.7
III	amygdala *	9.8	6.7	46.2	43.9	67.5	70.1	0.3	0.3	4.8	2.8	13.3	6.7
	fusiform *	5.5	5.5	31.8	35.6	62.3	61	0.3	0.3	2.1	2.8	0	0
	parahippocampal *	9.8	6.1	44.7	37.9	66.2	55.8	1	0.3	4.1	2.8	0	6.7
	lingual	1.8	2.5	12.1	15.9	35.1	31.2	0.7	0	0.7	0.7	0	0
IV	temporalpole	2.5	3.7	23.5	18.9	48.1	41.6	0.7	0.3	2.8	1.4	6.7	13.3
	inferiortemporal *	8.6	9.8	34.1	37.1	68.8	68.8	0.7	0.3	2.1	1.4	0	0
	middletemporal *	6.7	6.7	33.3	33.3	62.3	63.6	0.7	0.7	0	0.7	0	0
	isthmuscingulate	3.1	4.3	22	26.5	41.6	41.6	0.3	0	0.7	0.7	0	0
	caudalanteriorcingulate	1.8	1.8	7.6	5.3	11.7	13	0	0	0.7	0	0	0
	insula	4.3	2.5	16.7	16.7	35.1	28.6	0.7	0.7	1.4	2.1	0	0
	posteriorcingulate	3.1	2.5	19.7	17.4	35.1	31.2	0.7	0.3	1.4	1.4	0	0
	rostralanteriorcingulate	1.2	0.6	2.3	4.5	11.7	11.7	0	0	0.7	0	0	0
V_1	lateraloccipital	4.9	4.3	18.2	18.9	37.7	40.3	0.3	0	0.7	0	0	0
	inferiorparietal	4.9	6.1	22	27.3	54.5	49.4	0.3	0.7	0	0	0	0
	superiortemporal	4.3	1.8	17.4	17.4	33.8	27.3	0.7	0.3	0.7	0	0	0
	bankssts	7.4	2.5	25	19.7	51.9	44.2	0.7	0.3	0.7	0.7	0	0
	precuneus	2.5	4.3	15.9	18.9	37.7	41.6	0	0	0.7	0.7	0	0
	superiorparietal	4.3	4.3	14.4	14.4	41.6	36.4	0.3	0.3	0.7	1.4	0	0
	supramarginal	3.7	2.5	15.2	15.2	35.1	36.4	0.7	0.7	0	0	0	0
	transversetemporal	1.8	4.3	9.8	9.8	19.5	22.1	0.3	1.3	2.1	2.1	0	0
V_2	parsopercularis	1.8	1.2	11.4	10.6	26	23.4	0.7	0.7	0	1.4	0	0
	parsorbitalis	1.2	1.2	6.8	7.6	16.9	19.5	0	0	0	0	0	0
	parstriangularis	1.2	1.2	6.8	5.3	20.8	18.2	0.3	0.7	0	0	0	0
	frontalpole	1.2	0.6	3.8	4.5	15.6	16.9	0	0.3	0	0	0	0
	caudalmiddlefrontal	3.1	2.5	15.9	19.7	37.7	37.7	0.3	0.7	0.7	2.1	0	0
	lateralorbitofrontal	0.6	0.6	9.8	9.1	22.1	18.2	0	0	0.7	0.7	0	0
	medialorbitofrontal	1.2	0.6	4.5	8.3	14.3	18.2	0.3	0	0	1.4	0	0
	rostralmiddlefrontal	1.8	3.7	12.1	15.2	22.1	23.4	0	0	0	0	0	0
	superiorfrontal	1.8	2.5	8.3	9.8	23.4	23.4	0	0	0.7	0.7	0	0
VI	pericalcarine	0.6	0.6	6.8	7.6	23.4	15.6	0.3	0	0.7	0.7	0	0
	cuneus	1.8	1.2	12.9	11.4	31.2	28.6	0	0	0.7	0	0	0
	paracentral	3.1	1.8	6.1	4.5	18.2	13	0.7	0.3	2.8	1.4	0	0
	postcentral	1.8	2.5	4.5	3	16.9	20.8	0.3	1	0	0	0	0
	precentral	1.2	1.8	8.3	6.1	20.8	26	0.3	0.7	0.7	1.4	0	0

CU = Cognitively unimpaired, MCI = Mild cognitive impairment, AD = Alzheimer's disease, LH = Left hemisphere, RH = Right hemisphere, Aβ = Amyloid. V_2 represents Braak V region in the frontal lobe while V_1 regroups the remaining Braak V regions. An asterisk next to the region name indicate that this region is part of the temporal meta region of interest (Jack et al., 2017). Based on GMM-derived thresholds.

Supplementary Table 4 Regional tau abnormality progression across regions of interest

Braak stage	Region	CU Aβ+ (n = 90) LH	RH	MCI Aβ+ (n = 66) LH	RH	AD Aβ+ (n = 39) LH	RH	CU Aβ- (n = 96) LH	RH	MCI Aβ- (n = 40) LH	RH	AD Aβ- (n = 10) LH	RH
I	entorhinal *	8.9	14.4	12.1	4.5	10.3	5.1	3.1	1.0	5.0	2.5	0.0	0.0
III	amygdala *	3.3	6.7	10.6	9.1	2.6	0.0	1.0	1.0	2.5	2.5	10.0	0.0
	fusiform *	4.4	4.4	16.7	12.1	2.6	10.3	2.1	1.0	5.0	2.5	0.0	0.0
	parahippocampal *	7.8	7.8	6.1	13.6	5.1	5.1	1.0	3.1	2.5	5.0	10.0	0.0
	lingual	2.2	1.1	10.6	9.1	2.6	2.6	2.1	1.0	0.0	0.0	0.0	0.0
IV	temporalpole	6.7	5.6	12.1	16.7	12.8	15.4	1.0	1.0	2.5	0.0	10.0	0.0
	inferiortemporal *	4.4	4.4	12.1	13.6	2.6	7.7	2.1	2.1	2.5	7.5	0.0	0.0
	middletemporal *	5.6	6.7	12.1	12.1	5.1	2.6	1.0	1.0	2.5	0.0	0.0	0.0
	isthmuscingulate	4.4	2.2	10.6	4.5	7.7	5.1	1.0	2.1	0.0	0.0	0.0	0.0
	caudalanteriorcingulate	4.4	0.0	6.1	4.5	2.6	0.0	2.1	1.0	0.0	0.0	0.0	0.0
	insula	4.4	6.7	7.6	6.1	12.8	7.7	2.1	3.1	2.5	0.0	0.0	0.0
	posteriorcingulate	4.4	3.3	10.6	7.6	7.7	7.7	1.0	2.1	0.0	0.0	0.0	0.0
	rostralanteriorcingulate	2.2	1.1	3.0	3.0	5.1	0.0	2.1	1.0	0.0	0.0	0.0	0.0
V_1	lateraloccipital	2.2	2.2	12.1	9.1	5.1	7.7	1.0	1.0	2.5	2.5	0.0	0.0
	inferiorparietal	1.1	4.4	15.2	13.6	0.0	2.6	1.0	2.1	0.0	0.0	0.0	0.0
	superiortemporal	4.4	7.8	7.6	6.1	5.1	10.3	1.0	2.1	2.5	0.0	0.0	0.0
	bankssts	1.1	5.6	6.1	9.1	0.0	2.6	1.0	1.0	2.5	0.0	0.0	0.0
	precuneus	2.2	2.2	12.1	12.1	5.1	0.0	2.1	1.0	0.0	0.0	0.0	0.0
	superiorparietal	3.3	3.3	10.6	12.1	2.6	7.7	1.0	1.0	0.0	0.0	0.0	0.0
	supramarginal	4.4	4.4	10.6	12.1	12.8	2.6	1.0	1.0	0.0	0.0	0.0	0.0
	transversetemporal	4.4	1.1	3.0	9.1	10.3	2.6	2.1	3.1	0.0	0.0	0.0	0.0
V_2	parsopercularis	4.4	2.2	7.6	12.1	5.1	5.1	2.1	2.1	0.0	0.0	0.0	0.0
	parsorbitalis	2.2	1.1	3.0	0.0	2.6	0.0	2.1	3.1	0.0	0.0	0.0	0.0
	parstriangularis	2.2	1.1	1.5	9.1	5.1	0.0	2.1	2.1	0.0	0.0	0.0	0.0
	frontalpole	2.2	2.2	1.5	4.5	2.6	0.0	1.0	0.0	0.0	0.0	0.0	0.0
	caudalmiddlefrontal	3.3	7.8	7.6	4.5	2.6	2.6	2.1	0.0	0.0	0.0	0.0	0.0
	lateralorbitofrontal	3.3	1.1	7.6	7.6	0.0	0.0	2.1	2.1	0.0	0.0	0.0	0.0
	medialorbitofrontal	2.2	2.2	7.6	7.6	7.7	2.6	1.0	4.2	0.0	0.0	0.0	0.0
	rostralmiddlefrontal	4.4	5.6	4.5	3.0	7.7	2.6	2.1	2.1	0.0	0.0	0.0	0.0
	superiorfrontal	3.3	4.4	10.6	7.6	5.1	0.0	3.1	2.1	0.0	0.0	0.0	0.0
VI	pericalcarine	1.1	1.1	0.0	1.5	2.6	5.1	3.1	1.0	0.0	0.0	0.0	0.0
	cuneus	0.0	3.3	4.5	4.5	5.1	2.6	3.1	1.0	0.0	0.0	0.0	0.0
	paracentral	1.1	2.2	9.1	6.1	12.8	7.7	3.1	1.0	0.0	0.0	0.0	0.0
	postcentral	0.0	2.2	7.6	1.5	5.1	7.7	1.0	1.0	0.0	0.0	0.0	0.0
	precentral	2.2	4.4	7.6	7.6	2.6	5.1	2.1	1.0	0.0	0.0	0.0	0.0

Numbers are in percentages. CU = Cognitively unimpaired, MCI = Mild cognitive impairment, AD = Alzheimer's disease, LH = Left hemisphere, RH = Right hemisphere, Aβ = Amyloid. V_2 represents Braak V region in the frontal lobe while V_1 regroups the remaining Braak V regions. An asterisk next to the region name indicate that this region is part of the temporal meta region of interest (Jack et al., 2017). Based on GMM-derived thresholds.

Supplementary Table 5 Braak stages thresholds

Region	Threshold value<br>GMM-based	CU-based
Braak I	1.325	1.299
Braak III	1.359	1.274
Braak IV	1.347	1.249
Braak V	1.331	1.204
Braak VI	1.260	1.155
Temporal meta-ROI	1.398	1.311

Thresholds were derived by taking the composite regions and using the GMM method described in Figure 1, or using the mean plus two standard deviation of CU Aβ- participants
